# Red-Wine Gene Networks Linked to Exceptional Longevity in Humans

**DOI:** 10.3390/biom15101414

**Published:** 2025-10-04

**Authors:** Patricia Lacayo, Alexandria Martignoni, Kenneth Park, Christianne Castro, Shin Murakami

**Affiliations:** Department of Foundational Biomedical Sciences, College of Osteopathic Medicine, Touro University California, Vallejo, CA 94592, USA

**Keywords:** aging, longevity, centenarian, hormesis, alcohol, alternative medicine, polymorphism, red wine, polyphenol, resveratrol, catechins, rotundone, gallic acid

## Abstract

Despite the health concerns regarding alcohol and its link to cancer, moderate consumption of red wine has been associated with healthy aging and longevity, defined as up to one drink per day for women and two drinks per day for men (approximately 142 mL or 5 oz per drink). Previous research has revealed the health benefits of red wine, particularly in relation to cardiovascular disease. However, the influence of genetic factors on these benefits remains to be elucidated. In this study, we explored genes linked to red wine and created a curated gene set that intersects with those related to centenarians, which are markers of exceptional longevity. By analyzing literature from over 190 databases, we identified and validated a curated list of 43 genes associated with red wine and centenarians. We conducted gene set enrichment analysis as well as enrichment analysis of diseases and their tissue distributions. The results suggest that these genes play a crucial role in stress response and apoptosis, which are essential for cell survival and renewal. Additionally, these genes were enriched in pathways associated with smooth muscle cell proliferation, neuroinflammation, nucleotide excision repair, and lipoprotein metabolism (false discovery rate, FDR < 3 × 10^−7^). Gene set enrichment analysis indicated significant tissue distribution in the gastrointestinal, cardiovascular, and respiratory systems. Furthermore, the disease–gene enrichment analysis pointed to associations with diseases related to tissues and organs, including cardiovascular disease (heart disease and stroke), type 2 diabetes, gastrointestinal diseases and metabolic diseases, immune diseases, and cancer (FDR < 9.37 × 10^−6^); notably, cardiovascular diseases, diabetes, and cancer are leading causes of death, suggesting that these genes may be protective against those diseases. Our review of the literature indicates that individuals who do not currently drink alcohol should not be encouraged to start. However, we propose that moderate consumption of red wine, especially for middle-aged to older adults after 40 years old, can provide significant health benefits due to its components and the positive effects of hormesis. Although further research is necessary to uncover additional genes, this study provides the first genetic overview of the health benefits of red wine, emphasizing its potential in supporting healthy aging and longevity.

## 1. Introduction

The field of biological aging has made remarkable progress in uncovering the cellular and molecular mechanisms underlying age-related diseases [1,2,3]. Research on centenarian populations offers a unique perspective, guiding scientists in their efforts to identify factors that may promote longevity. However, a significant challenge persists: the relatively limited exploration of why centenarians demonstrate exceptional resistance to age-related diseases. A particularly intriguing phenomenon known as the French paradox [4,5] has garnered substantial scientific interest, particularly in light of the exceptional longevity exhibited by Jeanne Calment, a French centenarian who lived to the age of 122 years [6,7]. Epidemiological studies have consistently demonstrated that the French population has a lower incidence of coronary heart disease (CHD), a leading cause of death, despite a dietary pattern characterized by a higher intake of saturated fats [5]. This observation raises questions regarding the protective factors that may be implicated, with a notable emphasis on red wine consumption.

Recent studies have increasingly investigated the potential health benefits of red wine. Moderate consumption of red wine is associated with reduced risks of cardiovascular disease, cancer, and improved cognitive function. This level of moderate drinking is defined as one beverage daily for women and two beverages daily for men, with a standard drink measuring 142 mL (5 oz) [8,9,10]. Red wine can reduce inflammatory markers related to atherosclerosis in healthy individuals [11], inhibit aromatase, which may benefit patients with breast cancer [12], and enhance cognitive function when included in the diet [13]. Additionally, red wine has been found to lower markers of oxidative stress, inflammation, and nephropathy [14]. While previous studies have established the potential health benefits of red wine, the relationship between red wine and genetic factors has yet to be thoroughly explored. It is also worth noting that there is an ongoing debate regarding the health effects of alcohol (see Section 4).

Red wine contains a wide variety of biomolecules, such as catechins, rotundone, gallic acid, resveratrol (3,5,4′-trihydroxystilbene), and other polyphenols [15], which are known to interact with genes and gene products. Catechins are scavengers of reactive oxygen species and can inhibit pro-oxidant enzymes [16]. Resveratrol, a well-characterized component of red wine, targets SIRT1, a member of the Sirtuin family that encodes nicotinamide adenine dinucleotide-dependent deacetylase. Although SIRT1 function has yet to be identified in humans, sirtuin proteins in yeast regulate epigenetic gene silencing and suppress rDNA recombination [17]. SIRT1 is associated with reduced risks of hypertension, diabetes (i.e., reducing hemoglobin A1C), protective vascular function, metabolic syndrome, and cardiomyopathy [18,19]. Additionally, resveratrol increases the level of Sirtuin-1 by inhibiting the TLR4/NF-κB/STAT signal cascade and increases the level of brain-derived neurotrophic factor (BDNF) and NOS-3 (nitric oxide synthase-3) activity [20]. Red wine polyphenols suppress the secretion of ApoB (ApoB48 and ApoB100), affecting lipid metabolism [21,22]. Although mounting evidence suggests the health benefits of red wine, a red-wine component, resveratrol, may have no effects on cardiovascular health in clinical trials where the beneficial dose of resveratrol is much higher than the dose found in red wine [23].

A comprehensive genetic analysis of the health benefits associated with red wine is expected to enhance our understanding of its positive effects on health. In this study, we systematically investigated the genes that are upregulated or downregulated following the consumption of red wine and its components. Among the red wine genes, we identified genes associated with centenarians, serving as indicators of exceptional longevity. Our research aims to address existing knowledge gaps by compiling a gene set pertinent to red wine, delineating genetic networks linked to moderate red wine consumption, and elucidating the implications of these findings for human longevity and health benefits.

## 2. Materials and Methods

### 2.1. Study Design and Gene List Generation

Gene identification was performed as previously described [2,24,25,26]. Briefly, a comprehensive gene list was created using the keywords “red wine” and “centenarians” in the GeneCards program (www.genecards.org; last accessed 18 March 2025), which aggregates data from over 190 databases and references relevant scientific literature concerning human gene entries. We also used the keywords “white wine” and “centenarians.” The genes were ranked according to the Boolean model [27] to identify the corresponding documents, and the practical scoring function embedded in the GeneCards program was used to assess relevance. The retrieved data formed a primary gene list for further validation.

### 2.2. Gene Review and Validation

Three independent investigators reviewed the literature corresponding to each gene identified, and the consensus of the reviews was obtained. The inclusion criteria were as follows: (1) the literature was accessible; (2) each gene had been the focus of research in the cited studies; and (3) the result was statistically significant (*p* < 0.05). This ensured that only relevant studies were considered for analysis. The genes that exhibited inconsistencies across these reviews underwent a comprehensive validation process and were included only when the review demonstrated statistically significant outcomes (*p* < 0.05). Studies lacking confidence levels of *p*-values and/or any other statistical criteria, as well as those with inaccessible related research, were excluded from the analysis.

### 2.3. Artificial Intelligence (AI) Utilization for Literature Summarization

The use of a generative AI chatbot (Version ChatGPT-4o; https://chatgpt.org/; last accessed on 5 January 2025) was tested to evaluate its capacity to streamline the research. The information of each study manuscript, including the abstract, methods, and results, was used to generate summaries by the AI chatbot. These summaries were then reviewed, compared with the manual reviews, and validated for consistency with the study’s objectives. It is important to note that this process was conducted separately from the primary research and did not influence the study outcomes.

### 2.4. Data Analysis

We used multi-omics analyses, including Gene set enrichment analysis (GSEA), Network topology and over-representation (ORA) analyses, with the gene set generated in this study. GSEA was performed using STRING 12.5 (referred to as STRING-DB) [28] (https://string-db.org/, last accessed on 8 March 2025) as described previously [2,26,29]. Using STRING-DB, we performed Biological GO enrichment, tissue distribution enrichment, and disease association enrichment analysis. Network topology and ORA were performed using the web-based gene set analysis toolkit, WebGestalt 2024 [30] (https://www.webgestalt.org/, last accessed on 8 March 2025). Network Topology Analysis examines the characteristics of the identified genes, while ORA assesses the presence of genes in various categories, including biological, cellular, and molecular functions, as well as pathways (such as Reactome), tissues, and disease categories. The network database used was PPI BIOGRID [30]. We used statistical significance (*p*-value and false discovery rate, FDR), as described in the text.

### 2.5. Definition of Wine Consumption and Mid/Late Life

We define light and moderate red wine consumption as follows: Light red wine consumption is defined as one to three drinks per week; moderate red wine consumption refers to up to one drink per day for women and up to two drinks per day for men, with each drink being approximately 142 mL (5 oz) of red wine. Additionally, we define midlife as the period from ages 40 to 65, and late life as the period after the age of 65.

## 3. Results

### 3.1. Gene List Generation

**Figure 1:**
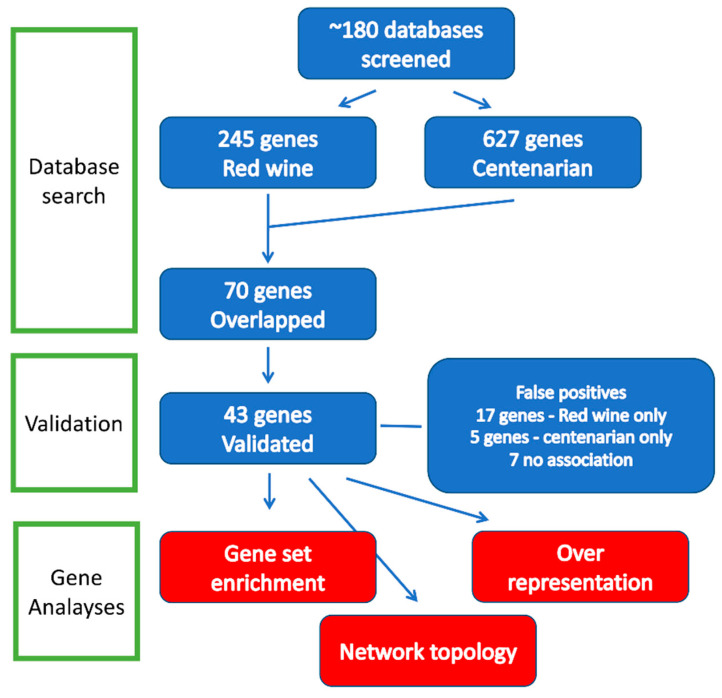
Flow diagram of gene identification and analysis. Three types of analyses, including network topology analysis, over-representation analysis (ORA), and gene set enrichment analysis (GSEA), were performed. See text for details.

Using approximately 190 databases [26], a total of 245 genes were found to be associated with the keyword “Red Wine,” while 627 genes were associated with “Centenarian.” This analysis identified 72 genes that were concurrently associated with both “Red Wine” and “Centenarian” (Section 2) (Figure 1). As a control, we searched for genes associated with the keywords “White Wine” and “Centenarian.” However, the search did not identify any genes in humans.

To confirm these findings, 72 genes were validated through 3 independent reviews. In cases where evidence was insufficient, such as instances of both upregulation and downregulation, a conservative statistical threshold of *p* < 0.05 was applied to establish significance. The validation process identified genes that did not meet the validation criteria and were categorized as false positives: five genes (6.9%) associated with red wine, seventeen genes (23.6%) associated with centenarians, and seven genes (9.7%) linked to both categories. We also validated our results using the AI chatbot, which identified 48 genes, including five false positives (resulting in a 10% error rate). In summary, we confirmed the existence of 43 genes associated with both red wine and centenarians, of which 40 were recognized by the analytical programs employed in this study (Section 2).

We conducted three types of analyses, as illustrated in Figure 1: network topology analysis, over-representation analysis (ORA), and gene set enrichment analysis (GSEA). Both ORA and GSEA evaluate the likelihood of overrepresentation within specific biological pathways. ORA calculates the number of genes from a given pathway included in a particular list, whereas GSEA analyzes the distribution of these pathway genes within a ranked list.

### 3.2. Network Topology Analysis and Over-Representation Analysis

**Figure 2:**
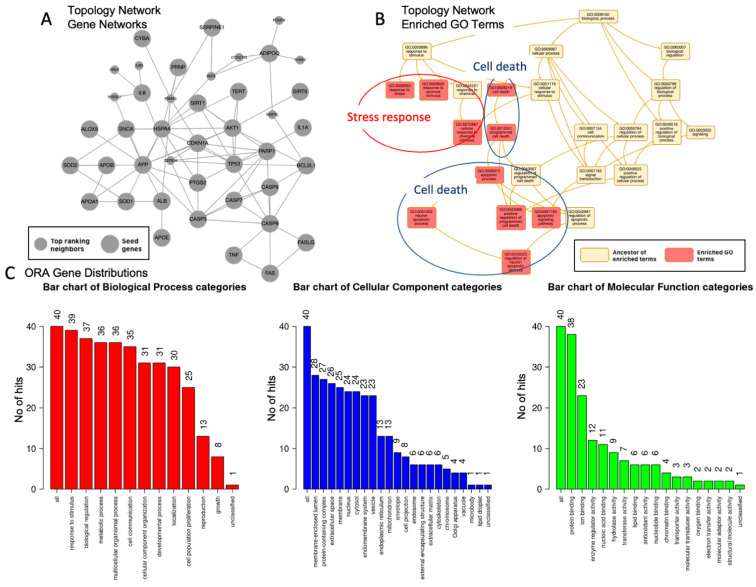
Nature of the genes associated with red wine and centenarians. (**A**). Network topology analysis of genes identified, showing a set of interactions within a single network. Seed genes (large gray nodes) represent the genes identified. Top-ranking neighbors (small gray nodes) represent the genes associated with the seed genes. (**B**). Network topology analysis of enriched GO terms (orange-pink, enriched GO terms; yellow, ancestors of the terms). The enriched GO terms are major categories that combine specific categories called ancestors of the terms, which can be summarized as two major categories: stress response (red circle) and cell death/ apoptosis (blue circle). (**C**). Over-presentation analysis (ORA) of the gene distributions, using gene ontology and the processes shown in the figure (red, biological category; cellular, cellular category; and green, molecular function categories). The numbers of each category are shown, and those with over 25 genes are listed in the text. Larger figures have been included.

Utilizing a threshold of false discovery rate (FDR) < 10 × 10^−5^, we conducted multiple analyses of the gene set, as shown in Figure 2. Figure 2A,B present the outcomes of the network topology analysis, while Figure 2C depicts gene over-representation by quantity. The analysis encompassed a search against 2194 genes within the networks, incorporating the top 10 neighboring genes in a single network (Figure 2A). The findings indicate an intricate interconnection among the analyzed genes, but not diverse genes unrelated to each other.

Figure 2B summarizes the enriched top 10 Gene Ontology categories, which are classified into two primary groups: (1) response to stress and stimuli, with an adjusted *p*-value < 4.2 × 10^−13^, and (2) apoptosis/cell death, with an adjusted *p*-value < 6.1 × 10^−13^. Figure 2C shows the gene distribution across three domains: (1) biological processes, comprising eight categories with over 25 genes per category, summarized as follows: response to stimulus (39 genes), biological functions (37 genes), metabolic processes (36 genes), cell communication (35 genes), cellular component organization (31 genes), and localization (30 genes); (2) cellular components, which includes four categories with over 25 genes per group, such as membrane-enriched lumen (28 genes), protein-containing complex (27 genes), extracellular space (26 genes), and membrane (25 genes); and (3) molecular function, characterized by a single category comprising over 25 genes, specifically protein binding (38 genes).

**Figure 3:**
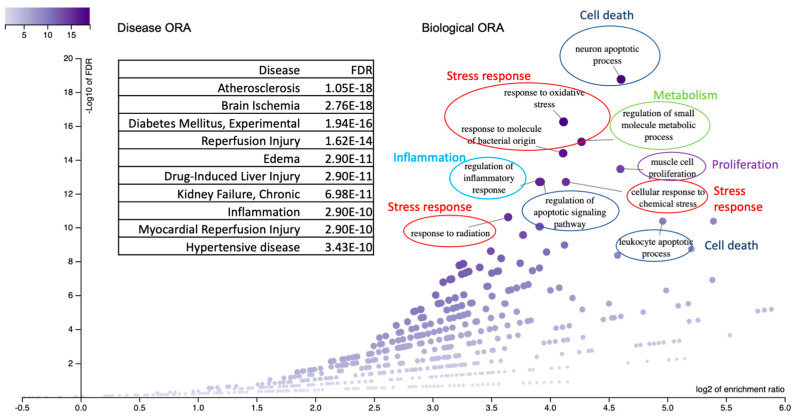
ORA of disease and biological categories is presented. The top 10 disease categories are labeled and displayed in the box with their respective statistics. The biological categories are represented in the dot plot, which is labeled accordingly. They include five major groups: cell death/apoptosis (blue circle), stress response (red circle), metabolism (green circle), proliferation (purple circle), and inflammation (pale blue circle).

Figure 3 shows an ORA of the disease enrichment analysis. The genes showed a significant enrichment with leading causes of death, including cardiovascular problems (arteriosclerosis, brain ischemia, reperfusion injury, hypertension, and other artery diseases), diabetes relevant to experimental systems, immune problems and inflammation, and kidney failure (FDR < 3.4 × 10^−10^). The list of diseases largely overlapped with the disease-enrichment results obtained by another method, gene set enrichment analysis (below). Figure 3 also shows an ORA of biological categories, which can be categorized into five major groups: 1. Apoptosis: This includes processes such as neuron apoptotic processes and leukocyte apoptotic processes. 2. Stress Response: This category encompasses responses to oxidative stress, bacterial stimuli, radiation, and chemical stress. 3. Metabolism: It is focused on the regulation of small-molecule (lipoprotein) metabolic processes. 4. Proliferation: This pertains to muscle cell proliferation. 5. Inflammation: Involves the regulation of the inflammatory response. It is worth noting that the response to bacterial stimuli is associated with the immune system.

### 3.3. Gene Set Enrichment Analysis

To further validate the results above, we used the GSEA to investigate biological pathways, tissue expressions, and disease–gene associations.

#### 3.3.1. Biological Pathways

**Figure 4:**
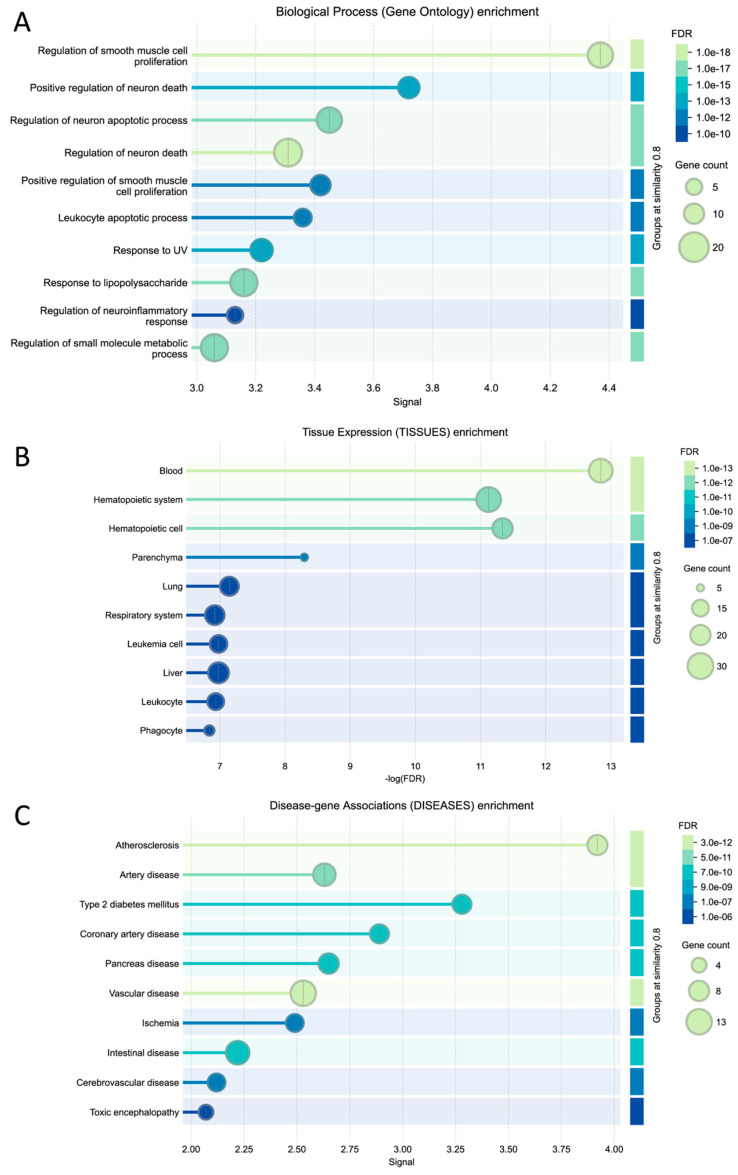
The GSEA result of the genes identified. (**A**). Biological ontology pathways, (**B**). Tissue expression profiles, and (**C**). Disease–gene association classifications. The top 10 categories were organized based on a similarity score of 0.8 or higher. A comprehensive discussion of the overall pathways is detailed in Section 4. Supplementary Material is available for a complete list of categories.

A total of 324 biological ontology pathways were identified (Table S1), with redundancies eliminated based on their similarity (refer to Section 2). Figure 4A presents a summary of the top 10 identified pathways, which include: (1) smooth muscle cell proliferation, recognized for its diverse roles across the gastrointestinal, cardiovascular, renal, and respiratory systems; (2) neuron-related pathways encompassing processes such as neural death and neuroinflammatory responses; (3) leukocyte apoptosis; (4) responses to ultraviolet light, specifically those involving nucleotide damage response and excision repair mechanisms; and (5) metabolic processes associated with lipoproteins. Notably, gene set enrichment analysis provided a more comprehensive understanding of the pathways compared to over-representation analysis, which primarily highlighted responses to stress and stimuli, as well as apoptosis and cell death. The GSEA biological pathways are consistent with the ORA biological categories (Figure 3).

#### 3.3.2. Tissue Distribution

To elucidate the sites of action of the identified genes, we conducted a comprehensive analysis of the enriched tissue categories. Our findings revealed a significant enrichment of gene expression in specific tissues associated with the cardiovascular system (notably the blood and hematopoietic systems), gastrointestinal tract (particularly the liver and digestive tissues), and respiratory system (including lung and related respiratory tissues) (FDR < 3 × 10^−7^). These results underscore the concentration of gene activity within tissues integral to key physiological functions, such as digestion, circulation, and respiration, thereby supporting the biological pathways previously delineated (refer to Section 4).

#### 3.3.3. Disease Association

Using disease association analysis, we identified 30 diseases exhibiting significant enrichment, with a false discovery rate (FDR) of less than 9.37 × 10^−6^ (Table 1). As illustrated in Table 1, the top ten diseases can be categorized as follows: (1) cardiovascular diseases, which represent heart disease and stroke, including atherosclerosis, vascular and arterial disease, and ischemic and cerebrovascular diseases, among others, with an FDR of less than 3.09 × 10^−7^; (2) type 2 diabetes, gastrointestinal diseases, and metabolic diseases, with an FDR of less than 9.24 × 10^−6^; (3) immune disease, with an FDR of less than 9.24 × 10^−6^; and (4) cancer, with an FDR of less than 1.04 × 10^−6^; and other diverse diseases; (4) cancer, with an FDR of less than 7.48 × 10^−6^ under the categories of cancer/neoplasm and tissue and other disease (Table 1).

## 4. Discussion

Most human traits are shaped by intricate interactions among multiple genes, known as complex traits, rather than solely by individual genes [31,32,33]. Gene network analysis is essential for mapping these complex interactions, significantly enhancing our understanding of their role in various phenotypic traits, including disease etiology. This study identified specific genes linked to red wine and centenarians that underpin its longevity benefits. We present evidence of biological pathways, tissue distributions, and disease associations that highlights the advantages of red wine and its connection to exceptional longevity.

### 4.1. Biological Pathways and Tissue Distributions

**Figure 5:**
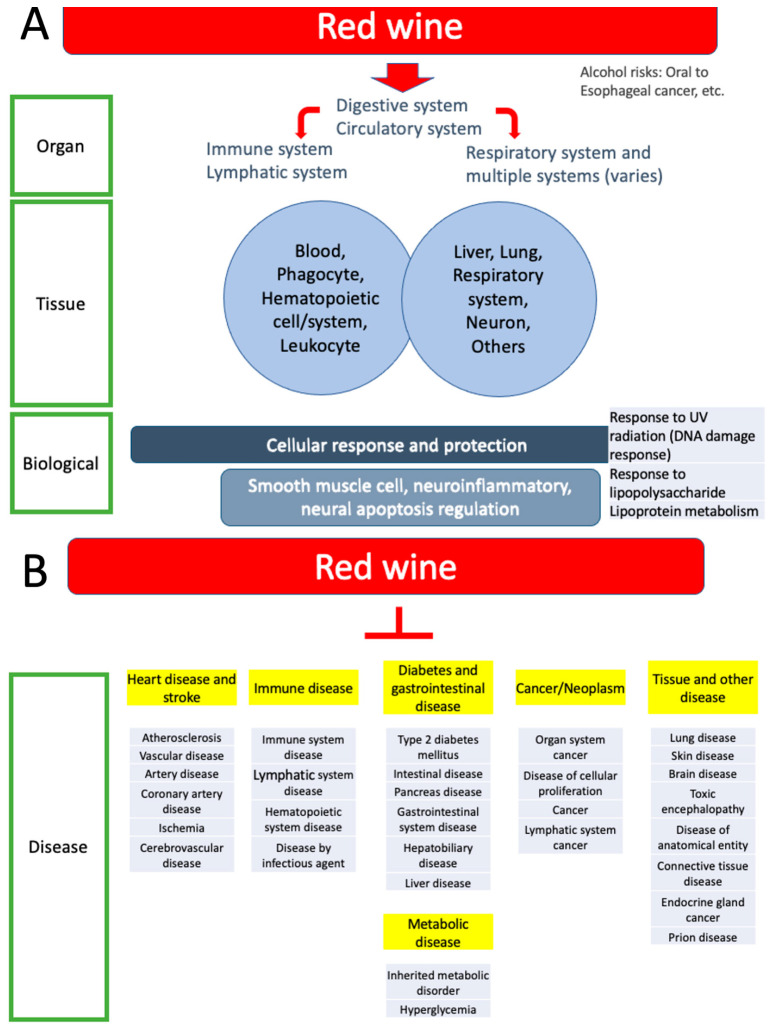
Summary of this study. (**A**). Organ systems, tissue expression enrichment, and biological enrichment are summarized. Most categories are included. Red wine is digested and absorbed in the digestive system, transported by the circulatory system, and exerts effects on various functions as indicated by the enrichment categories. See the discussion for risks of alcohol. (**B**). Disease association enrichment is organized into six groups (highlighted in yellow), including heart disease and stroke, immune disease, diabetes and gastrointestinal diseases, metabolic disease, cancer and neoplasm, tissue and other diseases (see also Table 1).

We used two methods to elucidate the hallmarks of centenarians and red wine. Figure 5 and Table 2 summarize the results of this study. The ORA analysis showed general processes, mainly comprising response to stimuli, cell death/apoptosis, and metabolic processes (Figure 2B,C). The GSGE analysis provided an overlap but a more detailed biological ontology pathway enriched among the red wine genes with a threshold of FDR < 10 × 10^−5^. Upon reducing redundancies among these pathways, four major biological hallmark groups were delineated (Figure 5A; Table 2), including (1) cell proliferation, including regulation of smooth muscle cells, (2) cell death, inflammation, and the immune system, including regulation of neuronal apoptosis/death and response to lipopolysaccharide, and regulation of neuroinflammatory responses, (3) stress response, including response to ultraviolet (UV) radiation (DNA damage response), and (4) metabolism including regulation of small-molecule metabolism (lipoprotein metabolism). In this study, we have combined cell death and inflammation and the immune system, since they are intertwined with each other.

The analysis of tissue distribution for these pathways revealed their essential roles across various biological systems, specifically highlighting three key areas: the gastrointestinal, cardiovascular, and respiratory systems (Figure 5A). Each of these systems plays a crucial role in the relationship between red wine consumption and longevity. In the gastrointestinal tract, the regulation of smooth muscle cells is vital for efficient food movement. In the cardiovascular system, smooth muscle cells play a crucial role as regulators of blood flow and blood pressure. Additionally, proper smooth muscle cell function is crucial for maintaining bronchioles in the respiratory system. This underscores the significance of smooth muscle regulation in various physiological contexts. Interestingly, there is an overlap between these tissues and the functions of the smooth muscle cells. Of the major sites of action [34], three overlap with the areas and functions of smooth muscle cells in the gastrointestinal tract (propulsion of the food bolus), cardiovascular system (regulation of blood flow and pressure through vascular resistance), and respiratory tract (regulation of bronchiole diameter).

### 4.2. Disease Associations

This study also revealed significant connections with a wide range of 30 disease categories, highlighting a low false discovery rate (FDR) of less than 9.37 × 10^−6^ (Table 1; Figure 5B). This level of precision suggests that the findings are robust. The study categorizes diseases into six groups and further confirms that cardiovascular diseases, particularly heart disease and stroke, are significantly prevalent (Table 1). Firstly, heart diseases refer to various health issues, including atherosclerosis, vascular disease, arterial disease, coronary artery disease, and ischemia. These conditions are also linked to stroke in the brain and cerebrovascular disease [35,36]. Notably, wine consumption is suggested to help protect against cognitive decline and cardiovascular diseases [34,35]. Additionally, previous studies suggest that consuming red wine, along with following a Mediterranean diet and making lifestyle changes, is linked to a lower risk of cognitive impairment, Alzheimer’s disease, and Parkinson’s disease [36,37,38,39,40,41,42], though a study suggests that drinking wine alone may not be effective in achieving these benefits [43].

Secondly, several categories, including diabetes, gastrointestinal diseases, and metabolic disorders, especially those affecting the pancreas and intestines, are closely interconnected and can be combined [44,45,46]. This suggests a potentially complex relationship in which red wine consumption may influence gut health and metabolic function, possibly due to the polyphenols found in red wine [47]. Thirdly, the results highlight various immune disease categories (Table 1). These include issues related to the hematopoietic, lymphatic, and immune systems, as well as blood cancers and infectious diseases [48]. Fourthly, the results identified a cancer category that emphasizes organ system and lymphatic system cancers. Consistently, a study suggests that drinking wine may lower the risk of cancer in the pancreas, skin, lung, and brain, as well as reduce the overall risk of cancer, showing no association with an increased risk of developing cancer [49]. Finally, the findings revealed tissue-specific diseases, including endocrine disorders and conditions affecting the liver, skin, brain, and connective tissues, all linked to the cardiovascular system. Notably, our results also included prion diseases, which are associated with protein misfolding. The results indicate a strong association that may support the idea that red wine consumption positively influences cardiovascular and associated tissue health by influencing genetic pathways, which could play a wide range of protective roles against these conditions.

### 4.3. Methodological Considerations and Limitations

We conducted a comprehensive search that led us to identify the genes linked to both red wine consumption and the phenomenon of centenarians. This study has a significant advantage due to its use of literature that has strict population controls, setting it apart from the observational studies commonly performed in alcohol epidemiological research. As a result, it provides an accurate and updated perspective on the health effects of red wine that may have been missed earlier. Methodological limitations are as follows. Firstly, upon closer examination, we discovered several discrepancies that highlighted the limitations of our study. Specifically, five of these genes (6.9%) were solely associated with red wine consumption. Additionally, we identified 17 genes (23.6%) that were exclusively related to the characteristics of centenarians. Further complicating the results, there were seven genes (9.7%) that showed no correlation with either red wine or centenarian status, leading to a total false-positive rate of 40.3%. We found that the AI chatbot, ChatGPT, showed 10% false positives, which was not accurate for validating the genes under the conditions used in this study. These findings underscore the necessity for rigorous validation of the text search methodology, as the current results may not accurately reflect the true associations. Secondly, another limitation was the likelihood that our gene list was not fully saturated, which may have missed molecular mechanisms. Further research may be needed to uncover additional relevant genes and ensure a more comprehensive understanding of the relationship between red wine and longevity.

Finally, the ORA, GSGA, and network topology methods have their advantages and limitations (Table 3) [30,50,51,52,53,54,55,56]. While the methods share the goal of identifying the biological significance of gene interactions, they differ in their approaches and the types of insights they provide. ORA focuses on determining whether a predefined set of genes is statistically overrepresented in a pool of genes of interest [30,50]. However, since ORA analyzes significant over-representations, it may overlook subtle changes. On the other hand, GSEA takes a more comprehensive approach by evaluating the entire ranked list of genes, rather than restricting the analysis to a specific subset [28,51]. While GSEA compares gene clusters for statistical significance, it may miss weaker associations among these clusters. The network topology analysis investigates changes in gene expression within established gene interaction networks, such as signaling and regulatory pathways. It emphasizes the topological relationships among genes and proteins, but its effectiveness depends on the accuracy and completeness of existing biological network information, which can be a notable limitation, particularly for newly discovered pathways. To address the limitations of these methods, we used both ORA and GSEA together, allowing us to gain a more complete understanding of the data. Our results showed the ORA analysis identified three biological ontology pathways (response to stimuli and cell death/apoptosis, and metabolic processes), which is advantageous for examining more specific categories. The GSGA analysis showed broader groups, summarized into six pathway groups, which can be advantageous for an overview of the biological categories. This is consistent with the concept of the methodology, in which ORA provides a targeted examination of specific gene sets, whereas GSEA offers a broader view of gene expression dynamics across all genes, highlighting the interconnectedness of different pathways and processes.

### 4.4. Considerations of Alcohol on Cancer

#### 4.4.1. Debate About Alcohol Consumption and Hormesis

The discussion about alcohol centers on two opposing views: one asserts that alcohol is unsafe at any dose, while the other claims that moderate consumption can offer health benefits. Firstly, alcohol has been classified as a Group 1 carcinogen by the International Agency for Research on Cancer (IARC) since 1987 [57]. A study conducted in the European Union found that light to moderate alcohol consumption, defined as up to 20 g per day (approximately 1.4 drinks of red wine), was linked to about 13.3% of alcohol-related cancer cases in 2017. This included around 11,000 cases of breast cancer among women [58]. The health effects of alcohol continue to be a topic of debate, claiming that any amount can be harmful [59,60]. In 2019, about 5% of alcohol drinkers in the United States were linked to an increased cancer risk; this risk significantly increases when combined with cigarette smoking, which has a strong positive correlation with alcohol consumption [61]. There are risks of oral cancer, esophageal cancer, breast cancer, and colorectal cancer by 1.04 to 1.8 times in light-to-moderate consumption [60]. Major health organizations, including the WHO and CDC, do not recommend drinking alcohol [59,60]. Thus, individuals who do not currently drink should not start; more details have been discussed elsewhere [60].

Secondly, safe levels of alcohol consumption vary significantly among different populations based on demographic and socioeconomic factors. Alcohol consumption of about 0.1 to 1.9 standard drinks per day, which is moderate consumption (Section 2.4), can result in better health outcomes, particularly for mid-to-older adults (aged 40 years and older) living in areas with high rates of cardiovascular diseases [62]. Conversely, for mid-to-younger adults (15–39 years old), the health benefits are associated with near-zero alcohol consumption [62]. Thus, we suggest that the health benefits of modest alcohol consumption begin as early as the age of 40. Additionally, it is known that socioeconomic status influences the risk of colorectal cancer [63]. While the number of observational studies examining these topics is on the rise, it is crucial to address the inequities among different subgroups, including age, location, genetics, modifiable risk factors (e.g., smoking/second-hand exposure to smoke, obesity, poor diet) [61], and socioeconomic factors. The genetic research employed in this study typically has extensive control over these variables, making it advantageous to interpret the results.

Furthermore, alcohol consumption of one to three drinks per week, which fall into light consumption (Section 2.4), has been associated with a lower risk of mortality compared to both non-drinkers and heavy drinkers in the mid-to-older population aged 55–74 years old (three or more drinks per day) [64]. The European Cooperation in Science and Technology (COST) 916 discussed the potential benefits of low-dose consumption of pure alcohol for lifespan [65]. Since then, there has been conflicting evidence regarding its impact on lifespan. A European randomized population study suggests that “alcohol does not provide any advantages for men or women and could shorten lifespan” [66]. In contrast, the Nederland cohort study indicates that the highest likelihood of reaching 90 years of age was associated with individuals who consumed 5–15 g of alcohol per day [67]. Thus, low doses of alcohol appear beneficial mid-to-late in life, with light-to-modest consumption (about three drinks per week) being the most effective. Taken together, there is evidence suggesting potential health benefits associated with low doses of alcohol when demographic differences are taken into account. The health benefits of red wine have been discussed in the Introduction and Section 4.4.2.

Hormesis refers to a biphasic adaptive response in which low doses of potentially harmful substances can provide benefits for survival and multiple stress resistance [68,69,70,71]. Previous research indicates a J-shaped relationship between alcohol intake and health risks in individuals aged 40 and older [62]. This dose–response relationship exemplifies the concept of hormesis. Notably, hormesis supports biological resilience, particularly in the context of aging and longevity [69,71,72]. We suggest that the health benefits associated with low alcohol consumption fall within the realm of hormesis, although these benefits appear to vary depending on the specific population. We emphasize that these potential benefits of alcohol consumption are currently a topic of discussion and debate.

#### 4.4.2. Debate About Wine Consumption

Red and white wine, particularly when consumed in moderation as part of a Mediterranean diet, differ from other alcoholic beverages and can offer health benefits without significantly increasing the risk of chronic diseases [42]. While both types of wine may have potential health advantages, red wine is often considered the healthier option due to its higher content of polyphenols, especially resveratrol and other phytochemicals [42,73]. These red wine components, as well as the hormesis of low-dose alcohol, have been discussed above (Section 1 and Section 4.4.1, respectively). Additionally, studies have linked white wine to an increased cancer risk in women [74], whereas red wine does not show a significant association with cancer risk (discussed below). Thus, there is a difference in the effects between red wine and white wine.

Moderate consumption of red wine (Section 2.4) does not elevate the risk of developing serious conditions such as Barrett’s esophagus (BE) and esophageal adenocarcinoma [75], prostate cancer [68,76], or stomach cancer [77]. Previous studies show that moderate red wine consumption either has no detrimental effects or actively reduces the risk of a broad range of cancers, including breast cancer, basal cell carcinoma, lung cancer, non-Hodgkin lymphoma, and colorectal cancer [10,37,38,78]. Consequently, red wine serves to counteract or safeguard against the risks of cancer. Furthermore, our studies have revealed significant associations with lymphatic cancer, endocrine cancer, and several cancer pathways. These compelling findings indicate that the genes linked to the effects of red wine play a crucial role in mitigating the harmful effects of toxic alcohol. The components of red wine (Section 1) and hormesis (Section 4.4.1) are expected to confer the health benefits.

## 5. Conclusions

This study examined the genetic factors linked to the health benefits of moderate red wine consumption, particularly its potential role in promoting longevity and healthy aging. It identified genes associated with centenarians that may protect against a variety of age-related diseases, including cardiovascular disease, diabetes, gastrointestinal diseases, metabolic disorders, and cancer. The analysis highlights the significance of these genes in key biological processes, such as stress response and tissue health.

The findings of this study suggest a genetic basis for the health benefits of red wine, indicating that moderate consumption can lead to a healthier lifespan. Our critical review of the available literature indicates that individuals who currently do not drink alcohol should not be advised to start (Section 4.4). However, we recommend that moderate red wine intake, particularly for those aged 40 and older, may offer substantial health advantages due to its beneficial components and the concept of hormesis (Section 4.4). We propose that the health advantages of moderate alcohol intake can start to emerge as early as age 40.

Additionally, the study emphasizes the need to consider demographic and socioeconomic factors in future research to assess the health benefits more accurately. It also highlights the importance of adopting a comprehensive and multidisciplinary approach to aging, focusing on the biological characteristics of centenarians and the effects of red wine to identify potential therapeutic strategies. Overall, the study reveals interesting associations that warrant further investigation, particularly to understand the underlying biological mechanisms and determine whether these associations can guide dietary recommendations or therapeutic approaches in preventing and managing various diseases.

## Figures and Tables

**Table 1:** Disease association with the red wine genes linked to centenarians. The top row indicates descriptions of each column. The major categories are highlighted in blue.

Term Description	Gene Hits	Control Gene Pool	FDR	Matching Proteins in Network
Heart disease and stroke				
Atherosclerosis	8	21	3.95 × 10^−12^	CCL2, APOB, APOA1, APOE, NOS3, IL6, ADIPOQ, TNF
Vascular disease	13	249	6.12 × 10^−12^	SERPINE1, CCL2, APOB, APOA1, APOE, ALB, NOS3, CASP3, MMP9, IL6, ADIPOQ, TNF, AKT1
Artery disease	10	130	1.85 × 10^−10^	CCL2, APOB, APOA1, APOE, ALB, NOS3, MMP9, IL6, ADIPOQ, TNF
Coronary artery disease	7	36	1.63 × 10^−9^	APOB, APOA1, APOE, ALB, NOS3, IL6, TNF
Ischemia	6	30	3.90 × 10^−8^	ALB, NOS3, CASP3, IL6, TNF, AKT1
Cerebrovascular disease	6	46	3.09 × 10^−8^	APOE, ALB, NOS3, CASP3, IL6, TNF
Diabetes and gastrointestinal disease				
Type 2 diabetes mellitus	7	25	2.17 × 10^−10^	APOE, ALB, IGF1, IL6, ADIPOQ, TNF, AKT1
Intestinal disease	11	216	5.83 × 10^−10^	CCL2, TP53, ALB, TERT, KLK3, MMP9, CDKN1A, IL6, TNF, AKT1, SNCA
Pancreas disease	8	70	1.82 × 10^−9^	TP53, TERT, CASP9, IGF1, IL6, ADIPOQ, TNF, AKT1
Gastrointestinal system disease	13	576	4.60 × 10^−8^	CCL2, TP53, ALB, TERT, CASP3, KLK3, MMP9, CDKN1A, IL6, TNF, AKT1, LDLR, SNCA
Hepatobiliary disease	8	168	6.79 × 10^−7^	TP53, ALB, TERT, CASP3, IL6, TNF, AKT1, LDLR
Liver disease	6	97	9.24 × 10^−6^	TP53, ALB, CASP3, IL6, TNF, AKT1
Metabolic disease				
Inherited metabolic disorder	14	949	1.06 × 10^−6^	APOB, APOA1, APOE, APP, ALB, IGF1, IL6, ADIPOQ, TNF, PRNP, AKT1, LDLR, ELANE, SNCA
Hyperglycemia	4	14	6.76 × 10^−6^	ALB, IL6, ADIPOQ, AKT1
Immune disease				
Immune system disease	17	675	1.55 × 10^−11^	CCL2, APOE, CYBA, IL1A, TP53, IL18, NOS3, CASP3, KLK3, ITGAL, CASP8, FASLG, BCL2L1, IL6, TNF, AKT1, FAS
Lymphatic system disease	8	174	8.18 × 10^−7^	TP53, CASP3, KLK3, CASP8, BCL2L1, TNF, AKT1, FAS
Hematopoietic system disease	10	473	6.71 × 10^−6^	GSR, TP53, CASP3, ITGAL, CASP8, BCL2L1, IL6, TNF, ELANE, FAS
Disease by infectious agent	9	368	9.24 × 10^−6^	APOE, TP53, APP, ALB, IL6, TNF, PRNP, LDLR, SNCA
Cancer/Neoplasm				
Organ system cancer	13	757	8.18 × 10^−7^	APOE, TP53, ALB, TERT, CASP3, KLK3, CASP8, BCL2L1, CDKN1A, IL6, TNF, AKT1, FAS
Disease of cellular proliferation	15	1101	8.18 × 10^−7^	APOE, TP53, ALB, TERT, CASP3, KLK3, SIRT6, CASP8, BCL2L1, IGF1, CDKN1A, IL6, TNF, AKT1, FAS
Cancer	14	978	1.40 × 10^−6^	APOE, TP53, ALB, TERT, CASP3, KLK3, SIRT6, CASP8, BCL2L1, CDKN1A, IL6, TNF, AKT1, FAS
Lymphatic system cancer	7	120	1.40 × 10^−6^	TP53, CASP3, CASP8, BCL2L1, TNF, AKT1, FAS
Tissue and other disease				
Lung disease	9	178	4.95 × 10^−8^	CCL2, CYBA, TP53, ALB, TERT, IL6, TNF, AKT1, ELANE
Skin disease	12	518	1.78 × 10^−7^	CCL2, APOA1, APOE, CYBA, IL1A, TP53, ALB, NOS3, TERT, CASP8, IGF1, TNF
Brain disease	13	806	1.40 × 10^−6^	APOE, TP53, SOD1, APP, ALB, NOS3, TERT, CASP3, MMP9, IL6, TNF, PRNP, SNCA
Toxic encephalopathy	4	9	1.79 × 10^−6^	APP, CASP3, IL6, SNCA
Disease of anatomical entity	34	4798	1.58 × 10^−11^	GSR, SERPINE1, CCL2, APOB, APOA1, APOE, CYBA, IL1A, TP53, SOD1, IL18, APP, ALB, NOS3, TERT, CASP3, KLK3, CASP9, ITGAL, CASP8, FASLG, MMP9, BCL2L1, IGF1, CDKN1A, IL6, ADIPOQ, TNF, PRNP, AKT1, LDLR, ELANE, FAS, SNCA
Connective tissue disease	12	774	6.76 × 10^−6^	CCL2, CYBA, IL1A, TP53, IL18, ALB, NOS3, TERT, MMP9, IL6, TNF, FAS
Endocrine gland cancer	6	93	7.48 × 10^−6^	APOE, TP53, ALB, TERT, CASP3, AKT1
Prion disease	4	16	9.37 × 10^−6^	APOE, APP, PRNP, SNCA

**Table 2:** A summary of categories identified by each method (see Section 2 and Section 4.1). The top row (gray) indicates categories and methods.

Categories	GSEA	ORA	Topology Network-GO Terms
Cell proliferation	Regulation of smooth muscle cells in the gastrointestinal, cardiovascular, renal, and respiratory systems)	Muscle cell proliferation	None
Cell death/apoptosis, inflammation and immune systems	Regulation of neuronal apoptosis/death (neural death and leukocyte apoptosis); Regulation of neuroinflammatory responses; Response to lipopolysaccharide	Neuron apoptotic process, regulation of apoptotic signaling pathway, and leukocyte apoptotic process; regulation of inflammatory response, response to molecule of bacterial origin (overlap with stress response)	Cell death, programmed cell death, apoptotic process, neuron apoptotic process, positive regulation of programmed cell death, apoptotic signaling pathway, regulation of neuron apoptotic process
Stress response	Response to ultraviolet (UV) radiation (DNA damage response)	Response to oxidative stress, response to molecule of bacterial origin, cellular response to chemical stress, response to radiation	Response to stress, response to external stimulus, cellular response to chemical stimulus
Metabolism	Regulation of small-molecule metabolism (lipoprotein metabolism)	Regulation of small molecule metabolic process (lipoprotein metabolism)	None

**Table 3:** A Summary of the methods used in this study (Section 2. Materials and Methods). The top row (gray) indicates methods and their explanations.

Method	Description	Advantages	Limitations
Over-Representation Analysis (ORA)	Assesses the proportion of genes within a pathway that are present among genes with differential expression.	Straightforward to interpret. Effective for pinpointing pathways that exhibit significant over-representations.	Highly sensitive to the user-specified cutoff for differentially expressed genes, which may overlook subtle changes. Also disregards the interactions among genes as well as quantitative data on gene expression.
Gene Set Enrichment Analysis (GSEA)	Evaluates if specific gene sets are concentrated at the extremes of a ranked gene list.	More sensitive than ORA, particularly in identifying subtle but coordinated alterations in pathways. It does not depend on random cutoffs.	May miss weaker associations among the clusters as well as possible cross-interaction between pathways.
Topology Network Analysis	Incorporates the framework of pathways into the evaluation, taking into account gene interactions.	Provides the most biological context and has the ability to reveal significant pathways that may overlook by taking gene-gene interactions into account.	Can be more complex than with ORA or GSEA and it relies on the accuracy and completeness of the reference network.

## Data Availability

All the data were made available within the manuscript.

## Supplementary Materials

The following supporting information can be downloaded at: https://www.mdpi.com/article/10.3390/biom15101414/s1, Table S1: Complete list of GSEA categories.

## Abbreviations

The following abbreviations are used in this manuscript:

AI	Artificial Intelligence
CHD	coronary heart disease
GSEA	Gene set enrichment analysis
ORA	Over-representation analysis
