# Supplementary material for: Red-Wine Gene Networks Linked to Exceptional Longevity in Humans
Source: Biomolecules. 2025 Oct 4;15(10):1414. doi: 10.3390/biom15101414 (PMC12562682; doi:10.3390/biom15101414)
Supplement: Supplementary file 1 [file biomolecules-15-01414-s001.zip › biomolecules-3815264-supplementary.pdf]

Supplementary Materials

Supplementary Table S1: Complete list of GSEA categories

Term description	Gene hits	Control gene pool	FDR	Matching proteins in network
Response to stress	39	3358	1.36E-22	SIRT1,GSR,SERPINE1,CCL2,APOE,CYBA,IL1A,TP53,SOD1,IL18,APP,ALB,NOS3,HSPA4,TERT,CASP3,CASP9,SIRT6,ITGAL,CASP8,PARP1,PTGS2,FASLG,CASP7,MMP9,ALOX5,BCL2L1,IGF1,CDKN1A,IL6,ADIPOQ,TNF,PRNP,SOD2,AKT1,LDLR,ELANE,FAS,SNCA
Regulation of response to stress	30	1373	9.98E-22	SIRT1,SERPINE1,APOA1,APOE,CYBA,IL1A,TP53,SOD1,IL18,APP,NOS3,KLK3,CASP9,SIRT6,CASP8,PARP1,PTGS2,MMP9,ALOX5,BCL2L1,IGF1,IL6,ADIPOQ,TNF,SOD2,AKT1,LDLR,ELANE,FAS,SNCA
Regulation of programmed cell death	30	1492	7.27E-21	SIRT1,SERPINE1,CCL2,APOE,IL1A,TP53,SOD1,APP,ALB,NOS3,TERT,CASP3,CASP9,CASP8,PARP1,PTGS2,FASLG,CASP7,MMP9,BCL2L1,IGF1,CDKN1A,IL6,ADIPOQ,TNF,PRNP,SOD2,AKT1,FAS,SNCA
Cellular response to chemical stimulus	35	2609	9.16E-21	SIRT1,GSR,SERPINE1,CCL2,APOB,APOE,CYBA,IL1A,TP53,SOD1,IL18,APP,ALB,NOS3,TERT,CASP3,CASP9,CASP8,PARP1,PTGS2,FASLG,CASP7,MMP9,ALOX5,BCL2L1,IGF1,IL6,ADIPOQ,TNF,PRNP,SOD2,AKT1,LDLR,FAS,SNCA
Response to oxygen-containing compound	30	1547	1.23E-20	SIRT1,SERPINE1,CCL2,APOB,APOE,CYBA,IL1A,TP53,SOD1,IL18,APP,NOS3,CASP3,CASP9,CASP8,PARP1,PTGS2,FASLG,MMP9,BCL2L1,IGF1,IL6,ADIPOQ,TNF,PRNP,SOD2,AKT1,LDLR,ELANE,SNCA
Regulation of apoptotic process	29	1462	5.74E-20	SIRT1,SERPINE1,CCL2,APOE,IL1A,TP53,SOD1,APP,ALB,NOS3,TERT,CASP3,CASP9,CASP8,PARP1,PTGS2,FASLG,CASP7,MMP9,BCL2L1,IGF1,IL6,ADIPOQ,TNF,PRNP,SOD2,AKT1,FAS,SNCA
Positive regulation of cell death	22	590	2.52E-19	SIRT1,CCL2,APOE,TP53,SOD1,APP,CASP3,CASP9,CASP8,PARP1,PTGS2,FASLG,CASP7,MMP9,CDKN1A,IL6,ADIPOQ,TNF,PRNP,SOD2,FAS,SNCA
Response to chemical	38	4010	4.98E-19	SIRT1,GSR,SERPINE1,CCL2,APOB,APOA1,APOE,CYBA,IL1A,TP53,SOD1,IL18,APP,ALB,NOS3,HSPA4,TERT,CASP3,CASP9,CASP8,PARP1,PTGS2,FASLG,CASP7,MMP9,ALOX5,BCL2L1,IGF1,IL6,ADIPOQ,TNF,PRNP,SOD2,AKT1,LDLR,ELANE,FAS,SNCA

Regulation of smooth muscle cell proliferation	15	142	2.09E-18	APOE,CYBA,IL18,NOS3,TERT,PTGS2,MMP9,IGF1,CDKN1A,IL6,ADIPOQ,TNF,SOD2,AKT1,ELANE
Regulation of neuron death	18	324	4.37E-18	SIRT1,CCL2,APOE,TP53,SOD1,APP,TERT,CASP3,CASP9,CASP8,PARP1,FASLG,BCL2L1,TNF,PRNP,SOD2,AKT1,SNCA
Response to abiotic stimulus	25	1107	9.23E-18	SIRT1,CYBA,IL1A,TP53,SOD1,APP,NOS3,TERT,CASP3,CASP9,SIRT6,CASP8,PARP1,PTGS2,CASP7,MMP9,BCL2L1,IGF1,CDKN1A,ADIPOQ,TNF,SOD2,AKT1,ELANE,FAS
Positive regulation of programmed cell death	20	519	1.33E-17	SIRT1,CCL2,TP53,SOD1,APP,CASP3,CASP9,CASP8,PTGS2,FASLG,CASP7,MMP9,CDKN1A,IL6,ADIPOQ,TNF,PRNP,SOD2,FAS,SNCA
Negative regulation of cell death	24	1016	2.58E-17	SIRT1,SERPINE1,CCL2,APOE,IL1A,TP53,SOD1,APP,ALB,NOS3,TERT,CASP3,CASP8,PTGS2,MMP9,BCL2L1,IGF1,IL6,TNF,PRNP,SOD2,AKT1,FAS,SNCA
Response to oxidative stress	18	368	2.84E-17	SIRT1,GSR,APOE,IL1A,TP53,SOD1,APP,NOS3,CASP3,PARP1,PTGS2,MMP9,IL6,ADIPOQ,PRNP,SOD2,AKT1,SNCA
Response to external stimulus	31	2355	3.25E-17	SIRT1,SERPINE1,CCL2,APOB,APOA1,APOE,CYBA,IL1A,TP53,SOD1,IL18,APP,ALB,NOS3,CASP3,CASP9,CASP8,PTGS2,FASLG,ALOX5,BCL2L1,CDKN1A,IL6,ADIPOQ,TNF,SOD2,AKT1,LDLR,ELANE,FAS,SNCA
Negative regulation of programmed cell death	23	911	4.50E-17	SIRT1,SERPINE1,CCL2,APOE,IL1A,TP53,SOD1,ALB,NOS3,TERT,CASP3,CASP8,PTGS2,MMP9,BCL2L1,IGF1,IL6,TNF,PRNP,SOD2,AKT1,FAS,SNCA
Cellular response to oxygen-containing compound	24	1057	4.93E-17	SIRT1,SERPINE1,CCL2,CYBA,IL1A,TP53,SOD1,IL18,APP,NOS3,CASP9,PARP1,PTGS2,MMP9,BCL2L1,IGF1,IL6,ADIPOQ,TNF,PRNP,SOD2,AKT1,LDLR,SNCA
Response to lipopolysaccharide	17	314	6.24E-17	SERPINE1,CCL2,APOB,IL1A,IL18,NOS3,CASP3,CASP9,CASP8,PTGS2,FASLG,IL6,TNF,SOD2,AKT1,ELANE,SNCA
Response to organic substance	32	2692	7.11E-17	SIRT1,SERPINE1,CCL2,APOB,CYBA,IL1A,TP53,SOD1,IL18,APP,NOS3,HSPA4,CASP3,CASP9,CASP8,PARP1,PTGS2,FASLG,CASP7,MMP9,BCL2L1,IGF1,IL6,ADIPOQ,TNF,PRNP,SOD2,AKT1,LDLR,ELANE,FAS,SNCA

Homeostatic process	26	1406	7.65E-17	SIRT1,GSR,CCL2,APOB,APOA1,APOE,CYBA,IL1A,SOD1,IL18,APP,ALB,NOS3,CASP3,SIRT6,PTGS2,FASLG,ALOX5,IL6,ADIPOQ,PRNP,SOD2,AKT1,LDLR,ELANE,FAS
Cellular response to organic substance	29	2019	1.14E-16	SIRT1,SERPINE1,CCL2,APOB,CYBA,IL1A,TP53,SOD1,IL18,APP,NOS3,CASP3,CASP9,CASP8,PARP1,PTGS2,FASLG,CASP7,BCL2L1,IGF1,IL6,ADIPOQ,TNF,PRNP,SOD2,AKT1,LDLR,FAS,SNCA
Regulation of small molecule metabolic process	17	330	1.15E-16	SIRT1,APOB,APOA1,APOE,TP53,SOD1,APP,NOS3,SIRT6,PARP1,PTGS2,IGF1,ADIPOQ,TNF,AKT1,LDLR,SNCA
Regulation of biological quality	35	3654	1.23E-16	SIRT1,GSR,SERPINE1,CCL2,APOB,APOA1,APOE,CYBA,IL1A,TP53,SOD1,IL18,APP,ALB,NOS3,TERT,CASP3,KLK3,SIRT6,PARP1,PTGS2,FASLG,ALOX5,BCL2L1,IGF1,IL6,ADIPOQ,TNF,PRNP,SOD2,AKT1,LDLR,ELANE,FAS,SNCA
Positive regulation of apoptotic process	19	507	1.35E-16	SIRT1,CCL2,TP53,SOD1,APP,CASP3,CASP9,CASP8,PTGS2,FASLG,CASP7,MMP9,IL6,ADIPOQ,TNF,PRNP,SOD2,FAS,SNCA
Regulation of neuron apoptotic process	15	216	2.89E-16	CCL2,APOE,TP53,SOD1,APP,TERT,CASP3,CASP9,PARP1,FASLG,BCL2L1,TNF,PRNP,SOD2,SNCA
Response to radiation	18	444	3.77E-16	SIRT1,CYBA,IL1A,TP53,APP,CASP3,CASP9,SIRT6,PARP1,PTGS2,CASP7,MMP9,BCL2L1,CDKN1A,TNF,SOD2,AKT1,ELANE
Negative regulation of apoptotic process	22	891	3.86E-16	SIRT1,SERPINE1,CCL2,APOE,IL1A,TP53,SOD1,ALB,NOS3,TERT,CASP3,PTGS2,MMP9,BCL2L1,IGF1,IL6,TNF,PRNP,SOD2,AKT1,FAS,SNCA
Regulation of catalytic activity	30	2370	3.92E-16	SIRT1,SERPINE1,CCL2,APOA1,APOE,CYBA,IL1A,TP53,SOD1,IL18,APP,NOS3,TERT,CASP3,CASP9,CASP8,PARP1,PTGS2,FASLG,MMP9,IGF1,CDKN1A,ADIPOQ,TNF,PRNP,SOD2,AKT1,ELANE,FAS,SNCA
Positive regulation of metabolic process	35	3847	5.26E-16	SIRT1,SERPINE1,CCL2,APOB,APOA1,APOE,CYBA,IL1A,TP53,SOD1,IL18,APP,NOS3,TERT,CASP3,KLK3,CASP9,SIRT6,CASP8,PARP1,PTGS2,FASLG,MMP9,IGF1,CDKN1A,IL6,ADIPOQ,TNF,PRNP,SOD2,AKT1,LDLR,ELANE,FAS,SNCA
Negative regulation of response to stimulus	26	1612	1.43E-15	SIRT1,SERPINE1,CCL2,APOA1,APOE,IL1A,TP53,SOD1,APP,NOS3,TERT,CASP8,PTGS2,MMP9,ALOX5,BCL2L1,IGF1,IL6,ADIPOQ,TNF,PRNP,SOD2,AKT1,LDLR,ELANE,SNCA

Regulation of developmental process	30	2492	1.47E-15	SIRT1,SERPINE1,CCL2,APOB,APOA1,APOE,IL1A,TP53,SOD1,IL18,APP,NOS3,TERT,KLK3,SIRT6,CASP8,PARP1,PTGS2,FASLG,MMP9,ALOX5,BCL2L1,IGF1,CDKN1A,IL6,ADIPOQ,TNF,SOD2,AKT1,LDLR
Response to lipid	21	827	1.63E-15	SERPINE1,CCL2,APOB,CYBA,IL1A,IL18,NOS3,CASP3,CASP9,CASP8,PARP1,PTGS2,FASLG,IL6,ADIPOQ,TNF,SOD2,AKT1,LDLR,ELANE,SNCA
Regulation of cell population proliferation	26	1669	3.05E-15	SIRT1,CCL2,APOE,CYBA,IL1A,TP53,IL18,APP,NOS3,TERT,CASP3,SIRT6,PTGS2,FASLG,MMP9,ALOX5,BCL2L1,IGF1,CDKN1A,IL6,ADIPOQ,TNF,PRNP,SOD2,AKT1,ELANE
Positive regulation of response to stimulus	28	2131	5.17E-15	SIRT1,SERPINE1,CCL2,APOA1,APOE,CYBA,IL1A,TP53,SOD1,IL18,APP,NOS3,TERT,KLK3,SIRT6,CASP8,PARP1,PTGS2,FASLG,MMP9,IGF1,IL6,ADIPOQ,TNF,LDLR,ELANE,FAS,SNCA
Regulation of defense response	19	638	5.98E-15	SERPINE1,APOA1,APOE,CYBA,SOD1,IL18,APP,KLK3,CASP8,PTGS2,MMP9,ALOX5,IGF1,IL6,ADIPOQ,TNF,LDLR,ELANE,SNCA
Response to inorganic substance	18	532	6.10E-15	SIRT1,APOB,IL1A,SOD1,APP,NOS3,TERT,CASP3,CASP9,CASP8,PARP1,PTGS2,MMP9,IL6,PRNP,SOD2,AKT1,SNCA
Positive regulation of macromolecule metabolic process	33	3533	8.49E-15	SIRT1,SERPINE1,CCL2,APOB,APOE,CYBA,IL1A,TP53,SOD1,IL18,APP,NOS3,TERT,CASP3,KLK3,CASP9,SIRT6,CASP8,PARP1,PTGS2,FASLG,MMP9,IGF1,CDKN1A,IL6,ADIPOQ,TNF,PRNP,AKT1,LDLR,ELANE,FAS,SNCA
Response to bacterium	19	663	1.10E-14	SERPINE1,CCL2,APOB,IL1A,IL18,APP,NOS3,CASP3,CASP9,CASP8,PTGS2,FASLG,IL6,ADIPOQ,TNF,SOD2,AKT1,ELANE,SNCA
Regulation of apoptotic signaling pathway	16	365	1.10E-14	SIRT1,SERPINE1,IL1A,TP53,SOD1,NOS3,TERT,PARP1,PTGS2,MMP9,BCL2L1,IGF1,TNF,SOD2,AKT1,FAS
Regulation of phosphate metabolic process	24	1405	1.29E-14	SIRT1,APOA1,APOE,TP53,SOD1,IL18,APP,NOS3,CASP3,SIRT6,PARP1,PTGS2,MMP9,IGF1,CDKN1A,IL6,ADIPOQ,TNF,PRNP,AKT1,LDLR,ELANE,FAS,SNCA
Positive regulation of molecular function	25	1587	1.29E-14	SIRT1,CCL2,APOA1,APOE,CYBA,SOD1,IL18,APP,NOS3,TERT,CASP9,CASP8,PARP1,FASLG,MMP9,IGF1,CDKN1A,IL6,ADIPOQ,TNF,PRNP,AKT1,ELANE,FAS,SNCA

Regulation of response to stimulus	34	3931	1.29E-14	SIRT1,SERPINE1,CCL2,APOA1,APOE,CYBA,IL1A,TP53,SOD1,IL18,APP,NOS3,TERT,KLK3,CASP9,SIRT6,CASP8,PARP1,PTGS2,FASLG,MMP9,ALOX5,BCL2L1,IGF1,IL6,ADIPOQ,TNF,PRNP,SOD2,AKT1,LDLR,ELANE,FAS,SNCA
Regulation of inflammatory response	16	371	1.29E-14	SERPINE1,APOA1,APOE,SOD1,IL18,APP,PTGS2,MMP9,ALOX5,IGF1,IL6,ADIPOQ,TNF,LDLR,ELANE,SNCA
Negative regulation of developmental process	21	933	1.29E-14	SIRT1,SERPINE1,APOE,IL1A,TP53,SOD1,IL18,APP,NOS3,KLK3,FASLG,MMP9,ALOX5,BCL2L1,IGF1,CDKN1A,IL6,ADIPOQ,TNF,SOD2,LDLR
Regulation of response to external stimulus	21	964	2.40E-14	SERPINE1,CCL2,APOA1,APOE,CYBA,SOD1,IL18,APP,NOS3,KLK3,CASP8,PTGS2,MMP9,ALOX5,IGF1,IL6,ADIPOQ,TNF,LDLR,ELANE,SNCA
Regulation of signaling	32	3367	2.43E-14	SIRT1,SERPINE1,CCL2,APOA1,APOE,CYBA,IL1A,TP53,SOD1,IL18,APP,NOS3,TERT,SIRT6,CASP8,PARP1,PTGS2,FASLG,MMP9,ALOX5,BCL2L1,IGF1,IL6,ADIPOQ,TNF,PRNP,SOD2,AKT1,LDLR,ELANE,FAS,SNCA
Regulation of molecular function	31	3085	2.71E-14	SIRT1,SERPINE1,CCL2,APOA1,APOE,CYBA,IL1A,TP53,SOD1,IL18,APP,NOS3,TERT,CASP3,CASP9,CASP8,PARP1,PTGS2,FASLG,MMP9,IGF1,CDKN1A,IL6,ADIPOQ,TNF,PRNP,SOD2,AKT1,ELANE,FAS,SNCA
Biological process involved in interspecies interaction between organisms	24	1490	4.06E-14	SERPINE1,CCL2,APOB,APOE,CYBA,IL1A,TP53,IL18,APP,NOS3,CASP3,CASP9,CASP8,PTGS2,FASLG,BCL2L1,IL6,ADIPOQ,TNF,SOD2,AKT1,LDLR,ELANE,SNCA
Positive regulation of cell communication	25	1693	4.69E-14	SIRT1,CCL2,APOA1,APOE,CYBA,IL1A,TP53,SOD1,IL18,APP,NOS3,TERT,SIRT6,CASP8,PARP1,PTGS2,FASLG,MMP9,IGF1,IL6,ADIPOQ,TNF,ELANE,FAS,SNCA
Positive regulation of signaling	25	1698	4.93E-14	SIRT1,CCL2,APOA1,APOE,CYBA,IL1A,TP53,SOD1,IL18,APP,NOS3,TERT,SIRT6,CASP8,PARP1,PTGS2,FASLG,MMP9,IGF1,IL6,ADIPOQ,TNF,ELANE,FAS,SNCA
Positive regulation of neuron death	11	92	4.93E-14	APOE,TP53,APP,CASP3,CASP9,CASP8,PARP1,FASLG,TNF,PRNP,SNCA
Cellular response to stimulus	39	6357	7.94E-14	SIRT1,GSR,SERPINE1,CCL2,APOB,APOA1,APOE,CYBA,IL1A,TP53,SOD1,IL18,APP,ALB,NOS3,TERT,CASP3,CASP9,SIRT6,ITGAL,CASP8,PARP1,PTGS2,FASLG

				,CASP7,MMP9,ALOX5,BCL2L1,IGF1,CDKN1A,IL6,ADIPOQ,TNF,PRNP,SOD2,AKT1,LDLR,FAS,SNCA
Positive regulation of catalytic activity	22	1191	8.13E-14	SIRT1,CCL2,APOA1,APOE,CYBA,SOD1,IL18,APP,NOS3,TERT,CASP9,CASP8,FASLG,IGF1,CDKN1A,ADIPOQ,TNF,PRNP,AKT1,ELANE,FAS,SNCA
Cellular response to chemical stress	14	272	1.32E-13	SIRT1,GSR,TP53,SOD1,NOS3,CASP3,PARP1,PTGS2,MMP9,IL6,SOD2,AKT1,FAS,SNCA
Response to UV	12	150	1.49E-13	SIRT1,TP53,CASP3,CASP9,SIRT6,PARP1,PTGS2,CASP7,MMP9,CDKN1A,AKT1,ELANE
Programmed cell death	21	1084	1.97E-13	SIRT1,IL1A,TP53,SOD1,APP,CASP3,CASP9,CASP8,PARP1,FASLG,CASP7,MMP9,BCL2L1,CDKN1A,IL6,TNF,SOD2,AKT1,ELANE,FAS,SNCA
Response to cytokine	19	804	2.33E-13	SIRT1,CCL2,APOB,CYBA,IL1A,TP53,IL18,APP,CASP3,CASP8,PTGS2,FASLG,BCL2L1,IL6,ADIPOQ,TNF,AKT1,FAS,SNCA
Regulation of immune system process	23	1438	2.43E-13	SIRT1,SERPINE1,CCL2,APOA1,APOE,CYBA,IL1A,SOD1,IL18,APP,CASP3,KLK3,CASP8,IGF1,CDKN1A,IL6,ADIPOQ,TNF,PRNP,AKT1,LDLR,ELANE,SNCA
Regulation of cell communication	31	3355	2.45E-13	SIRT1,SERPINE1,CCL2,APOA1,APOE,CYBA,IL1A,TP53,SOD1,IL18,APP,NOS3,TERT,SIRT6,CASP8,PARP1,PTGS2,FASLG,MMP9,ALOX5,BCL2L1,IGF1,IL6,ADIPOQ,TNF,PRNP,SOD2,AKT1,ELANE,FAS,SNCA
Positive regulation of cellular process	37	5584	2.53E-13	SIRT1,SERPINE1,CCL2,APOB,APOA1,APOE,CYBA,IL1A,TP53,SOD1,IL18,APP,NOS3,TERT,CASP3,CASP9,SIRT6,CASP8,PARP1,PTGS2,FASLG,CASP7,MMP9,ALOX5,BCL2L1,IGF1,CDKN1A,IL6,ADIPOQ,TNF,PRNP,SOD2,AKT1,LDLR,ELANE,FAS,SNCA
Response to stimulus	41	7835	2.57E-13	SIRT1,GSR,SERPINE1,CCL2,APOB,APOA1,APOE,CYBA,IL1A,TP53,SOD1,IL18,APP,ALB,NOS3,HSPA4,TERT,CASP3,CASP9,SIRT6,ITGAL,CASP8,PARP1,PTGS2,FASLG,CASP7,MMP9,ALOX5,BCL2L1,IGF1,CDKN1A,IL6,ADIPOQ,TNF,PRNP,SOD2,AKT1,LDLR,ELANE,FAS,SNCA
Response to organonitrogen compound	20	963	3.18E-13	SIRT1,CYBA,IL1A,TP53,SOD1,APP,CASP3,PARP1,PTGS2,CASP7,MMP9,BCL2L1,IGF1,IL6,ADIPOQ,TNF,PRNP,AKT1,LDLR,SNCA
Regulation of localization	26	2103	4.28E-13	SIRT1,SERPINE1,CCL2,APOB,APOA1,APOE,CYBA,IL1A,SOD1,APP,NOS3,TERT,SIRT6,PARP1,PTGS2,FASLG,MMP9,ALOX5,BCL2L1,IGF1,IL6,ADIPOQ,TNF,PRNP,AKT1,SNCA

Positive regulation of gene expression	21	1146	5.08E-13	SIRT1,SERPINE1,APOB,CYBA,IL1A,TP53,SOD1,IL18,APP,NOS3,TERT,KLK3,CASP8,PTGS2,IGF1,IL6,ADIPOQ,TNF,AKT1,LDLR,ELANE
Positive regulation of biological process	38	6207	5.52E-13	SIRT1,SERPINE1,CCL2,APOB,APOA1,APOE,CYBA,IL1A,TP53,SOD1,IL18,APP,NOS3,TERT,CASP3,KLK3,CASP9,SIRT6,CASP8,PARP1,PTGS2,FASLG,CASP7,MMP9,ALOX5,BCL2L1,IGF1,CDKN1A,IL6,ADIPOQ,TNF,PRNP,SOD2,AKT1,LDLR,ELANE,FAS,SNCA
Response to other organism	22	1328	6.07E-13	SERPINE1,CCL2,APOB,CYBA,IL1A,TP53,IL18,APP,NOS3,CASP3,CASP9,CASP8,PTGS2,FASLG,BCL2L1,IL6,ADIPOQ,TNF,SOD2,AKT1,ELANE,SNCA
Positive regulation of protein metabolic process	23	1512	6.10E-13	SIRT1,APOE,TP53,IL18,APP,CASP3,CASP9,SIRT6,CASP8,PTGS2,FASLG,MMP9,IGF1,CDKN1A,IL6,ADIPOQ,TNF,PRNP,AKT1,LDLR,ELANE,FAS,SNCA
Response to organic cyclic compound	19	861	6.41E-13	CCL2,APOB,CYBA,IL1A,SOD1,IL18,APP,CASP3,CASP9,CASP8,PARP1,PTGS2,CASP7,BCL2L1,IL6,ADIPOQ,TNF,SOD2,SNCA
Negative regulation of biological process	36	5313	6.41E-13	SIRT1,SERPINE1,CCL2,APOA1,APOE,CYBA,IL1A,TP53,SOD1,IL18,APP,ALB,NOS3,TERT,CASP3,KLK3,SIRT6,CASP8,PARP1,PTGS2,FASLG,MMP9,ALOX5,BCL2L1,IGF1,CDKN1A,IL6,ADIPOQ,TNF,PRNP,SOD2,AKT1,LDLR,ELANE,FAS,SNCA
Regulation of intracellular signal transduction	24	1726	7.32E-13	SIRT1,CCL2,APOA1,APOE,IL1A,TP53,SOD1,IL18,APP,CASP8,PARP1,PTGS2,FASLG,MMP9,BCL2L1,IGF1,IL6,ADIPOQ,TNF,PRNP,SOD2,AKT1,ELANE,FAS
Negative regulation of signaling	22	1354	8.30E-13	SIRT1,SERPINE1,APOA1,APOE,IL1A,TP53,APP,NOS3,TERT,CASP8,PTGS2,MMP9,BCL2L1,IGF1,IL6,ADIPOQ,TNF,PRNP,SOD2,AKT1,LDLR,SNCA
Response to endogenous stimulus	22	1363	9.38E-13	SIRT1,CCL2,APOB,CYBA,TP53,SOD1,APP,NOS3,CASP3,CASP9,PARP1,PTGS2,CASP7,BCL2L1,IGF1,IL6,ADIPOQ,TNF,PRNP,AKT1,LDLR,SNCA
Positive regulation of smooth muscle cell proliferation	10	85	1.07E-12	CYBA,IL18,TERT,PTGS2,MMP9,IGF1,IL6,TNF,AKT1,ELANE
Apoptotic process	20	1041	1.10E-12	SIRT1,IL1A,TP53,SOD1,APP,CASP3,CASP9,CASP8,PARP1,FASLG,CASP7,MMP9,BCL2L1,CDKN1A,IL6,TNF,SOD2,AKT1,FAS,SNCA

Regulation of transport	24	1763	1.10E-12	SIRT1,SERPINE1,CCL2,APOA1,APOE,CYBA,IL1A,SOD1,APP,NOS3,TERT,SIRT6,PTGS2,FASLG,MMP9,ALOX5,BCL2L1,IGF1,IL6,ADIPOQ,TNF,PRNP,AKT1,SNCA
Regulation of signal transduction	29	2978	1.12E-12	SIRT1,SERPINE1,CCL2,APOA1,APOE,CYBA,IL1A,TP53,SOD1,IL18,APP,NOS3,TERT,CASP8,PARP1,PTGS2,FASLG,MMP9,BCL2L1,IGF1,IL6,ADIPOQ,TNF,PRNP,SOD2,AKT1,ELANE,FAS,SNCA
Regulation of cell differentiation	23	1582	1.35E-12	SIRT1,SERPINE1,APOB,APOA1,IL1A,TP53,SOD1,IL18,APP,TERT,SIRT6,CASP8,PARP1,PTGS2,MMP9,ALOX5,IGF1,IL6,ADIPOQ,TNF,SOD2,AKT1,LDLR
Chemical homeostasis	19	904	1.35E-12	SIRT1,APOB,APOA1,APOE,CYBA,IL1A,SOD1,IL18,APP,SIRT6,FASLG,ALOX5,IL6,ADIPOQ,PRNP,SOD2,AKT1,LDLR,ELANE
Positive regulation of transport	19	915	1.60E-12	SIRT1,SERPINE1,CCL2,APOA1,APOE,CYBA,IL1A,SOD1,APP,TERT,SIRT6,PTGS2,FASLG,IGF1,ADIPOQ,TNF,PRNP,AKT1,SNCA
Regulation of multicellular organismal process	28	2749	1.60E-12	SIRT1,SERPINE1,APOA1,APOE,CYBA,IL1A,TP53,SOD1,IL18,APP,NOS3,TERT,KLK3,SIRT6,CASP8,PARP1,PTGS2,FASLG,MMP9,ALOX5,IGF1,IL6,ADIPOQ,TNF,PRNP,AKT1,LDLR,ELANE
Regulation of lipid metabolic process	14	346	2.08E-12	SIRT1,APOB,APOA1,APOE,IL1A,SOD1,APP,SIRT6,PTGS2,ADIPOQ,TNF,AKT1,LDLR,SNCA
Regulation of phosphorylation	21	1251	2.15E-12	SIRT1,APOA1,APOE,TP53,SOD1,IL18,APP,CASP3,SIRT6,PTGS2,MMP9,IGF1,CDKN1A,IL6,ADIPOQ,TNF,PRNP,AKT1,ELANE,FAS,SNCA
Negative regulation of signal transduction	21	1252	2.16E-12	SIRT1,SERPINE1,APOA1,APOE,IL1A,TP53,APP,NOS3,TERT,CASP8,PTGS2,MMP9,BCL2L1,IGF1,IL6,ADIPOQ,TNF,PRNP,SOD2,AKT1,SNCA
Response to tumor necrosis factor	12	199	2.33E-12	SIRT1,CCL2,APOB,CYBA,TP53,CASP3,CASP8,PTGS2,ADIPOQ,TNF,AKT1,FAS
Negative regulation of cellular process	34	4736	2.34E-12	SIRT1,SERPINE1,CCL2,APOA1,APOE,IL1A,TP53,SOD1,IL18,APP,ALB,NOS3,TERT,CASP3,SIRT6,CASP8,PARP1,PTGS2,FASLG,MMP9,ALOX5,BCL2L1,IGF1,CDKN1A,IL6,ADIPOQ,TNF,PRNP,SOD2,AKT1,LDLR,ELANE,FAS,SNCA
Regulation of protein phosphorylation	20	1108	3.04E-12	SIRT1,APOA1,APOE,TP53,SOD1,IL18,APP,CASP3,PTGS2,MMP9,IGF1,CDKN1A,IL6,ADIPOQ,TNF,PRNP,AKT1,ELANE,FAS,SNCA
Response to metal ion	14	362	3.50E-12	IL1A,SOD1,APP,TERT,CASP3,CASP9,CASP8,PARP1,PTGS2,MMP9,PRNP,SOD2,AKT1,SNCA

Positive regulation of small molecule metabolic process	11	147	3.50E-12	SIRT1,APOA1,APOE,APP,NOS3,PTGS2,IGF1,ADIPOQ,TNF,AKT1,SNCA
Response to nutrient levels	15	461	3.86E-12	SIRT1,APOE,CYBA,IL1A,TP53,SOD1,ALB,PTGS2,CDKN1A,ADIPOQ,TNF,SOD2,AKT1,LDLR,FAS
Positive regulation of developmental process	21	1332	6.67E-12	SIRT1,SERPINE1,APOB,APOA1,APOE,IL1A,IL18,APP,NOS3,TERT,SIRT6,CASP8,PARP1,PTGS2,ALOX5,IGF1,IL6,ADIPOQ,TNF,SOD2,AKT1
Positive regulation of signal transduction	22	1525	7.22E-12	SIRT1,CCL2,APOA1,APOE,CYBA,IL1A,TP53,SOD1,IL18,APP,NOS3,TERT,CASP8,PARP1,FASLG,MMP9,IGF1,IL6,ADIPOQ,TNF,ELANE,FAS
Negative regulation of apoptotic signaling pathway	12	230	1.09E-11	SIRT1,SERPINE1,IL1A,NOS3,TERT,PTGS2,MMP9,BCL2L1,IGF1,TNF,SOD2,AKT1
Regulation of cytokine production	17	739	1.10E-11	SIRT1,SERPINE1,APOA1,CYBA,IL1A,SOD1,IL18,APP,CASP8,PTGS2,ALOX5,IGF1,IL6,ADIPOQ,TNF,PRNP,ELANE
Negative regulation of multicellular organismal process	19	1035	1.19E-11	SERPINE1,APOA1,APOE,CYBA,TP53,SOD1,APP,NOS3,KLK3,PTGS2,FASLG,ALOX5,IGF1,IL6,ADIPOQ,TNF,PRNP,LDLR,ELANE
Cellular response to stress	22	1572	1.26E-11	SIRT1,GSR,IL1A,TP53,SOD1,ALB,NOS3,TERT,CASP3,CASP9,SIRT6,PARP1,PTGS2,MMP9,BCL2L1,CDKN1A,IL6,TNF,SOD2,AKT1,FAS,SNCA
Response to light stimulus	13	314	1.38E-11	SIRT1,TP53,APP,CASP3,CASP9,SIRT6,PARP1,PTGS2,CASP7,MMP9,CDKN1A,AKT1,ELANE
Apoptotic signaling pathway	13	318	1.60E-11	SIRT1,IL1A,TP53,CASP3,CASP9,CASP8,FASLG,CASP7,BCL2L1,CDKN1A,TNF,SOD2,FAS
Circulatory system development	18	901	1.61E-11	SIRT1,SERPINE1,CCL2,APOB,APOE,IL1A,TP53,IL18,NOS3,CASP3,SIRT6,CASP8,PTGS2,CASP7,CDKN1A,SOD2,AKT1,LDLR
Leukocyte apoptotic process	8	45	2.48E-11	TP53,CASP3,CASP9,FASLG,BCL2L1,IL6,AKT1,FAS
Regulation of cell activation	16	658	3.00E-11	CCL2,APOE,IL1A,SOD1,IL18,APP,NOS3,CASP3,IGF1,CDKN1A,IL6,TNF,PRNP,AKT1,LDLR,SNCA

Positive regulation of cellular metabolic process	28	3114	3.02E-11	SIRT1,APOA1,APOE,CYBA,IL1A,TP53,SOD1,IL18,APP,NOS3,TERT,CASP3,SIRT6,PARP1,PTGS2,MMP9,IGF1,CDKN1A,IL6,ADIPOQ,TNF,PRNP,SOD2,AKT1,LDLR,ELANE,FAS,SNCA
Cellular response to endogenous stimulus	19	1103	3.39E-11	SIRT1,CCL2,APOB,CYBA,TP53,SOD1,APP,CASP3,CASP9,PARP1,PTGS2,CASP7,BCL2L1,IGF1,ADIPOQ,TNF,PRNP,AKT1,SNCA
Positive regulation of nitrogen compound metabolic process	28	3166	4.51E-11	SIRT1,APOE,IL1A,TP53,IL18,APP,NOS3,TERT,CASP3,CASP9,SIRT6,CASP8,PARP1,PTGS2,FASLG,MMP9,IGF1,CDKN1A,IL6,ADIPOQ,TNF,PRNP,SOD2,AKT1,LDLR,ELANE,FAS,SNCA
Regulation of protein metabolic process	26	2622	4.82E-11	SIRT1,SERPINE1,APOA1,APOE,TP53,SOD1,IL18,APP,CASP3,CASP9,SIRT6,CASP8,PTGS2,FASLG,MMP9,IGF1,CDKN1A,IL6,ADIPOQ,TNF,PRNP,AKT1,LDLR,ELANE,FAS,SNCA
Positive regulation of multicellular organismal process	21	1505	5.98E-11	SIRT1,SERPINE1,APOE,CYBA,IL1A,SOD1,IL18,APP,NOS3,TERT,SIRT6,CASP8,PARP1,PTGS2,ALOX5,IGF1,IL6,ADIPOQ,TNF,AKT1,ELANE
Cellular response to organonitrogen compound	15	574	7.08E-11	CYBA,TP53,SOD1,APP,CASP3,PARP1,PTGS2,CASP7,BCL2L1,IGF1,ADIPOQ,TNF,PRNP,AKT1,SNCA
Regulation of cysteine-type endopeptidase activity involved in apoptotic process	11	205	8.96E-11	SIRT1,APP,CASP9,CASP8,PTGS2,FASLG,MMP9,TNF,AKT1,FAS,SNCA
Regulation of macromolecule metabolic process	36	6249	9.17E-11	SIRT1,SERPINE1,CCL2,APOB,APOA1,APOE,CYBA,IL1A,TP53,SOD1,IL18,APP,NOS3,TERT,CASP3,KLK3,CASP9,SIRT6,CASP8,PARP1,PTGS2,FASLG,MMP9,ALOX5,IGF1,CDKN1A,IL6,ADIPOQ,TNF,PRNP,SOD2,AKT1,LDLR,ELANE,FAS,SNCA
Negative regulation of cell population proliferation	16	713	9.18E-11	CCL2,APOE,IL1A,TP53,APP,NOS3,CASP3,SIRT6,PTGS2,ALOX5,CDKN1A,IL6,ADIPOQ,TNF,PRNP,SOD2
Regulation of metabolic process	37	6784	1.15E-10	SIRT1,SERPINE1,CCL2,APOB,APOA1,APOE,CYBA,IL1A,TP53,SOD1,IL18,APP,NOS3,TERT,CASP3,KLK3,CASP9,SIRT6,CASP8,PARP1,PTGS2,FASLG,MMP9,A

				LOX5,BCL2L1,IGF1,CDKN1A,IL6,ADIPOQ,TNF,PRNP,SOD2,AKT1,LDLR,ELANE,FAS,SNCA
Regulation of locomotion	18	1032	1.37E-10	SIRT1,SERPINE1,CCL2,APOE,IL1A,APP,NOS3,TERT,PTGS2,MMP9,IGF1,IL6,ADIPOQ,TNF,SOD2,AKT1,ELANE,SNCA
Positive regulation of cell differentiation	17	876	1.37E-10	SIRT1,SERPINE1,APOB,APOA1,IL18,APP,TERT,SIRT6,CASP8,PARP1,PTGS2,IGF1,IL6,ADIPOQ,TNF,SOD2,AKT1
Regulation of multicellular organismal development	20	1389	1.49E-10	SIRT1,SERPINE1,IL1A,TP53,SOD1,IL18,NOS3,TERT,KLK3,SIRT6,CASP8,FASLG,MMP9,ALOX5,IGF1,IL6,ADIPOQ,TNF,AKT1,LDLR
Regulation of proteolysis	16	739	1.50E-10	SIRT1,SERPINE1,APOE,TP53,APP,CASP9,SIRT6,CASP8,PTGS2,FASLG,MMP9,TNF,PRNP,AKT1,FAS,SNCA
Positive regulation of protein phosphorylation	16	747	1.75E-10	SIRT1,TP53,IL18,APP,PTGS2,MMP9,IGF1,CDKN1A,IL6,ADIPOQ,TNF,PRNP,AKT1,ELANE,FAS,SNCA
Negative regulation of gene expression	17	899	1.98E-10	SIRT1,SERPINE1,APOA1,APOE,TP53,APP,TERT,SIRT6,IGF1,CDKN1A,IL6,ADIPOQ,TNF,PRNP,AKT1,LDLR,ELANE
Cellular response to oxidative stress	11	224	2.07E-10	SIRT1,GSR,TP53,SOD1,NOS3,PARP1,MMP9,IL6,SOD2,AKT1,SNCA
Positive regulation of phosphate metabolic process	17	912	2.44E-10	SIRT1,TP53,IL18,APP,NOS3,PTGS2,MMP9,IGF1,CDKN1A,IL6,ADIPOQ,TNF,PRNP,AKT1,ELANE,FAS,SNCA
Negative regulation of catalytic activity	16	771	2.69E-10	SIRT1,SERPINE1,APOA1,APOE,TP53,APP,NOS3,CASP3,PTGS2,MMP9,CDKN1A,ADIPOQ,TNF,PRNP,AKT1,SNCA
Multicellular organismal process	36	6490	2.87E-10	SIRT1,SERPINE1,CCL2,APOB,APOA1,APOE,CYBA,IL1A,TP53,SOD1,IL18,APP,ALB,NOS3,CASP3,KLK3,CASP9,SIRT6,CASP8,PARP1,PTGS2,FASLG,CASP7,MMP9,ALOX5,BCL2L1,IGF1,CDKN1A,IL6,ADIPOQ,TNF,PRNP,SOD2,AKT1,LDLR,SNCA
Regulation of neuroinflammatory response	7	34	2.95E-10	IL18,PTGS2,MMP9,IGF1,IL6,TNF,LDLR

Regulation of cell migration	17	927	3.01E-10	SIRT1,SERPINE1,CCL2,APOE,IL1A,APP,NOS3,TERT,PTGS2,MMP9,IGF1,IL6,ADIPOQ,TNF,SOD2,AKT1,ELANE
Regulation of endopeptidase activity	13	414	3.20E-10	SIRT1,SERPINE1,APP,CASP9,CASP8,PTGS2,FASLG,MMP9,TNF,PRNP,AKT1,FAS,SNCA
Response to oxygen levels	12	318	3.20E-10	SIRT1,CYBA,IL1A,TP53,TERT,CASP3,PTGS2,ADIPOQ,TNF,SOD2,AKT1,FAS
Regulation of lipid localization	10	167	3.42E-10	SIRT1,APOB,APOA1,APOE,IL1A,FASLG,IL6,ADIPOQ,TNF,AKT1
Response to reactive oxygen species	10	169	3.80E-10	SIRT1,APOE,IL1A,SOD1,NOS3,CASP3,MMP9,IL6,SOD2,AKT1
Immune system process	23	2121	3.85E-10	SIRT1,CCL2,CYBA,IL1A,TP53,SOD1,IL18,APP,CASP3,CASP9,ITGAL,CASP8,PARP1,FASLG,MMP9,ALOX5,IL6,TNF,SOD2,AKT1,ELANE,FAS,SNCA
Cellular response to abiotic stimulus	12	325	3.94E-10	SIRT1,CYBA,TP53,CASP3,CASP9,CASP8,PARP1,PTGS2,MMP9,BCL2L1,CDKN1A,FAS
Inflammatory response	14	538	4.31E-10	CCL2,CYBA,IL1A,IL18,APP,ITGAL,PTGS2,CASP7,ALOX5,IL6,TNF,AKT1,ELANE,SNCA
Regulation of lipid biosynthetic process	10	172	4.33E-10	SIRT1,APOB,APOA1,APOE,IL1A,SOD1,PTGS2,TNF,AKT1,LDLR
System development	29	3867	6.09E-10	SIRT1,SERPINE1,CCL2,APOB,APOA1,APOE,IL1A,TP53,SOD1,IL18,APP,NOS3,CASP3,CASP9,SIRT6,CASP8,PARP1,PTGS2,FASLG,CASP7,MMP9,BCL2L1,IGF1,CDKN1A,IL6,TNF,SOD2,AKT1,LDLR
Regulation of hydrolase activity	17	1011	1.04E-09	SIRT1,SERPINE1,CCL2,APOA1,SOD1,APP,NOS3,CASP9,CASP8,PTGS2,FASLG,MMP9,TNF,PRNP,AKT1,FAS,SNCA
Regulation of cellular response to stress	15	712	1.10E-09	SIRT1,IL1A,TP53,SOD1,APP,CASP9,SIRT6,PARP1,PTGS2,ALOX5,BCL2L1,TNF,SOD2,AKT1,FAS
Cell surface receptor signaling pathway	22	2040	1.57E-09	SIRT1,CCL2,APOA1,IL1A,TP53,IL18,APP,NOS3,CASP3,ITGAL,CASP8,PARP1,FASLG,CASP7,MMP9,BCL2L1,IGF1,IL6,TNF,AKT1,FAS,SNCA
Regulation of cell-cell adhesion	13	480	1.70E-09	CCL2,APOA1,IL1A,IL18,CASP3,ALOX5,IGF1,IL6,ADIPOQ,TNF,PRNP,AKT1,ELANE

Regulation of fat cell differentiation	9	135	1.70E-09	SIRT1,SIRT6,PTGS2,ALOX5,IL6,ADIPOQ,TNF,SOD2,AKT1
Regulation of cellular response to oxidative stress	8	84	1.72E-09	SIRT1,SOD1,APP,PARP1,ALOX5,TNF,SOD2,AKT1
Positive regulation of cytokine production	13	482	1.76E-09	SIRT1,SERPINE1,CYBA,IL1A,SOD1,IL18,APP,CASP8,PTGS2,IL6,ADIPOQ,TNF,ELANE
Regulation of angiogenesis	11	288	2.27E-09	SIRT1,SERPINE1,IL1A,NOS3,TERT,KLK3,SIRT6,FASLG,ALOX5,IL6,TNF
Negative regulation of response to external stimulus	12	387	2.51E-09	SERPINE1,CCL2,APOA1,APOE,SOD1,NOS3,ALOX5,IGF1,ADIPOQ,TNF,LDLR,ELANE
Response to decreased oxygen levels	11	291	2.51E-09	SIRT1,CYBA,IL1A,TP53,TERT,CASP3,PTGS2,ADIPOQ,TNF,SOD2,AKT1
Regulation of transferase activity	16	912	2.58E-09	SIRT1,APOA1,APOE,TP53,SOD1,IL18,APP,CASP3,IGF1,CDKN1A,ADIPOQ,TNF,PRNP,AKT1,ELANE,SNCA
Response to ionizing radiation	9	143	2.62E-09	SIRT1,CYBA,IL1A,TP53,CASP3,PARP1,BCL2L1,CDKN1A,SOD2
Regulation of reactive oxygen species metabolic process	9	143	2.62E-09	CYBA,TP53,SOD1,APP,ALOX5,CDKN1A,TNF,SOD2,SNCA
Neuron apoptotic process	8	91	2.96E-09	TP53,APP,CASP3,CASP9,CASP7,BCL2L1,FAS,SNCA
Cellular response to organic cyclic compound	13	508	3.15E-09	CCL2,CYBA,SOD1,IL18,APP,CASP3,CASP9,CASP8,PTGS2,CASP7,ADIPOQ,TNF,SNCA
Negative regulation of neuron death	10	219	3.64E-09	SIRT1,CCL2,APOE,SOD1,APP,TERT,BCL2L1,SOD2,AKT1,SNCA

Regulation of gene expression	31	4899	3.81E-09	SIRT1,SERPINE1,APOB,APOA1,APOE,CYBA,IL1A,TP53,SOD1,IL18,APP,NOS3,TERT,KLK3,SIRT6,CASP8,PARP1,PTGS2,FASLG,ALOX5,IGF1,CDKN1A,IL6,ADIPOQ,TNF,PRNP,SOD2,AKT1,LDLR,ELANE,SNCA
Positive regulation of cell population proliferation	16	945	4.14E-09	SIRT1,CYBA,IL1A,IL18,TERT,SIRT6,PTGS2,FASLG,MMP9,BCL2L1,IGF1,CDKN1A,IL6,TNF,AKT1,ELANE
Positive regulation of neuron apoptotic process	7	54	4.34E-09	TP53,APP,CASP3,CASP9,FASLG,TNF,PRNP
Regulation of biosynthetic process	29	4205	4.44E-09	SIRT1,CCL2,APOB,APOA1,APOE,CYBA,IL1A,TP53,SOD1,IL18,APP,NOS3,TERT,SIRT6,PARP1,PTGS2,FASLG,ALOX5,IGF1,CDKN1A,IL6,ADIPOQ,TNF,PRNP,SOD2,AKT1,LDLR,ELANE,SNCA
Animal organ development	26	3246	4.52E-09	SIRT1,SERPINE1,CCL2,APOA1,IL1A,TP53,SOD1,IL18,APP,NOS3,CASP3,CASP9,SIRT6,CASP8,PARP1,PTGS2,FASLG,CASP7,MMP9,BCL2L1,IGF1,CDKN1A,IL6,TNF,SOD2,AKT1
Negative regulation of extrinsic apoptotic signaling pathway	8	97	4.52E-09	SERPINE1,IL1A,NOS3,TERT,BCL2L1,IGF1,TNF,AKT1
Response to gamma radiation	7	55	4.74E-09	CYBA,IL1A,TP53,PARP1,BCL2L1,CDKN1A,SOD2
Regulation of steroid metabolic process	8	98	4.78E-09	SIRT1,APOB,APOA1,APOE,IL1A,SOD1,TNF,LDLR
Positive regulation of cell migration	13	529	4.78E-09	SIRT1,SERPINE1,IL1A,APP,NOS3,TERT,PTGS2,MMP9,IGF1,IL6,TNF,SOD2,AKT1
Positive regulation of lipid metabolic process	9	155	4.78E-09	APOA1,APOE,IL1A,APP,PTGS2,ADIPOQ,TNF,AKT1,LDLR
Regulation of protein kinase activity	14	663	5.22E-09	SIRT1,APOE,SOD1,IL18,APP,CASP3,IGF1,CDKN1A,ADIPOQ,TNF,PRNP,AKT1,ELANE,SNCA

Negative regulation of metabolic process	25	2982	5.28E-09	SIRT1,SERPINE1,APOA1,APOE,TP53,SOD1,APP,TERT,CASP3,SIRT6,PARP1,PTGS2,FASLG,MMP9,BCL2L1,IGF1,CDKN1A,IL6,ADIPOQ,TNF,PRNP,AKT1,LDLR,ELANE,SNCA
Negative regulation of phosphate metabolic process	12	424	6.13E-09	SIRT1,APOE,TP53,CASP3,SIRT6,PARP1,CDKN1A,ADIPOQ,TNF,PRNP,AKT1,SNCA
Negative regulation of small molecule metabolic process	8	104	7.18E-09	SIRT1,APOE,TP53,SOD1,SIRT6,PARP1,ADIPOQ,AKT1
Intrinsic apoptotic signaling pathway	9	166	8.13E-09	SIRT1,TP53,CASP3,CASP9,CASP7,BCL2L1,CDKN1A,TNF,SOD2
Positive regulation of intracellular signal transduction	16	997	8.18E-09	SIRT1,CCL2,APOA1,APOE,IL1A,TP53,SOD1,IL18,APP,CASP8,FASLG,IGF1,IL6,ADIPOQ,TNF,ELANE
Response to peptide	12	437	8.28E-09	SIRT1,CYBA,TP53,APP,PARP1,PTGS2,MMP9,IGF1,ADIPOQ,TNF,PRNP,AKT1
Regulation of vascular associated smooth muscle cell proliferation	7	61	8.57E-09	TERT,MMP9,IGF1,CDKN1A,ADIPOQ,TNF,SOD2
Regulation of intrinsic apoptotic signaling pathway	9	169	9.21E-09	SIRT1,TP53,SOD1,PARP1,PTGS2,MMP9,BCL2L1,SOD2,AKT1
Regulation of protein localization	15	852	1.04E-08	APOE,IL1A,APP,TERT,SIRT6,PARP1,PTGS2,ALOX5,BCL2L1,IGF1,IL6,ADIPOQ,TNF,PRNP,AKT1
Defense response	18	1394	1.19E-08	SERPINE1,CCL2,CYBA,IL1A,TP53,IL18,APP,ITGAL,PTGS2,FASLG,CASP7,ALOX5,BCL2L1,IL6,TNF,AKT1,ELANE,SNCA
Cellular response to toxic substance	8	114	1.35E-08	GSR,APOE,SOD1,ALB,NOS3,PTGS2,TNF,SOD2
Cell communication	31	5165	1.38E-08	SIRT1,CCL2,APOA1,APOE,IL1A,TP53,SOD1,IL18,APP,ALB,NOS3,CASP3,CASP9,ITGAL,CASP8,PARP1,PTGS2,FASLG,CASP7,MMP9,BCL2L1,IGF1,CDKN1A,IL6,ADIPOQ,TNF,PRNP,SOD2,AKT1,FAS,SNCA

Negative regulation of protein metabolic process	16	1038	1.39E-08	SIRT1,SERPINE1,APOE,TP53,APP,CASP3,PTGS2,MMP9,IGF1,CDKN1A,ADIPOQ,TNF,PRNP,AKT1,LDLR,SNCA
Positive regulation of transferase activity	13	586	1.47E-08	SIRT1,APOA1,APOE,IL18,APP,IGF1,CDKN1A,ADIPOQ,TNF,PRNP,AKT1,ELANE,SNCA
Cellular response to radiation	9	183	1.73E-08	SIRT1,CYBA,TP53,CASP9,PARP1,PTGS2,MMP9,BCL2L1,CDKN1A
Positive regulation of reactive oxygen species metabolic process	7	69	1.80E-08	CYBA,TP53,SOD1,APP,CDKN1A,SOD2,SNCA
Regulation of leukocyte activation	13	601	1.96E-08	CCL2,IL1A,SOD1,IL18,CASP3,IGF1,CDKN1A,IL6,TNF,PRNP,AKT1,LDLR,SNCA
Regulation of cellular component organization	22	2365	2.11E-08	SIRT1,SERPINE1,APOA1,APOE,CYBA,IL1A,TP53,APP,SIRT6,PARP1,FASLG,MMP9,BCL2L1,IGF1,CDKN1A,IL6,ADIPOQ,TNF,PRNP,AKT1,LDLR,SNCA
Regulation of leukocyte cell-cell adhesion	11	368	2.20E-08	CCL2,IL1A,IL18,CASP3,ALOX5,IGF1,IL6,TNF,PRNP,AKT1,ELANE
Regulation of oxidative stress-induced cell death	7	73	2.55E-08	SIRT1,SOD1,APP,PARP1,TNF,SOD2,AKT1
Positive regulation of proteolysis	11	375	2.65E-08	SIRT1,APOE,APP,CASP9,SIRT6,CASP8,FASLG,TNF,AKT1,FAS,SNCA
Response to hypoxia	10	278	2.79E-08	SIRT1,CYBA,IL1A,TP53,TERT,CASP3,PTGS2,ADIPOQ,TNF,SOD2
Negative regulation of nitrogen compound metabolic process	22	2403	2.80E-08	SIRT1,SERPINE1,APOE,TP53,APP,TERT,CASP3,SIRT6,PARP1,PTGS2,FASLG,MMP9,IGF1,CDKN1A,IL6,ADIPOQ,TNF,PRNP,AKT1,LDLR,ELANE,SNCA
Intrinsic apoptotic signaling pathway in	7	75	2.99E-08	SIRT1,TP53,CASP9,BCL2L1,CDKN1A,TNF,SOD2

response to DNA damage				
Cellular homeostasis	13	628	3.19E-08	SIRT1,GSR,CCL2,APOE,CYBA,IL1A,SOD1,APP,NOS3,FASLG,IL6,PRNP,ELANE
Positive regulation of cysteine-type endopeptidase activity involved in apoptotic process	8	129	3.19E-08	SIRT1,APP,CASP9,CASP8,FASLG,TNF,FAS,SNCA
Regulation of cell adhesion	14	784	3.81E-08	SERPINE1,CCL2,APOA1,IL1A,IL18,CASP3,ALOX5,IGF1,IL6,ADIPOQ,TNF,PRNP,AKT1,ELANE
Regulation of cellular catabolic process	14	789	4.11E-08	SIRT1,APOA1,APOE,TP53,APP,CASP3,SIRT6,BCL2L1,IGF1,IL6,TNF,AKT1,LDLR,SNCA
Positive regulation of catabolic process	12	513	4.35E-08	SIRT1,APOA1,APOE,APP,CASP3,SIRT6,IGF1,IL6,TNF,AKT1,LDLR,SNCA
Positive regulation of defense response	10	296	4.81E-08	SERPINE1,CYBA,IL18,APP,KLK3,PTGS2,IL6,TNF,LDLR,SNCA
Regulation of endothelial cell proliferation	8	138	5.10E-08	SIRT1,CCL2,APOE,CYBA,SIRT6,ALOX5,TNF,AKT1
Anatomical structure morphogenesis	21	2229	5.10E-08	SIRT1,SERPINE1,CCL2,APOB,APOE,TP53,SOD1,IL18,APP,NOS3,CASP3,CASP9,CASP8,PTGS2,FASLG,MMP9,BCL2L1,IL6,TNF,AKT1,LDLR
Negative regulation of macromolecule metabolic process	23	2760	5.20E-08	SIRT1,SERPINE1,APOA1,APOE,TP53,APP,TERT,CASP3,SIRT6,PARP1,PTGS2,FASLG,MMP9,IGF1,CDKN1A,IL6,ADIPOQ,TNF,PRNP,AKT1,LDLR,ELANE,SNCA
Signaling	30	5057	5.22E-08	SIRT1,CCL2,APOA1,APOE,IL1A,TP53,SOD1,IL18,APP,NOS3,CASP3,CASP9,ITGAL,CASP8,PARP1,PTGS2,FASLG,CASP7,MMP9,BCL2L1,IGF1,CDKN1A,IL6,ADIPOQ,TNF,PRNP,SOD2,AKT1,FAS,SNCA
Regulation of primary metabolic process	32	5899	6.25E-08	SIRT1,SERPINE1,APOB,APOA1,APOE,IL1A,TP53,SOD1,IL18,APP,NOS3,TERT,CASP3,CASP9,SIRT6,CASP8,PARP1,PTGS2,FASLG,MMP9,IGF1,CDKN1A,IL6,ADIPOQ,TNF,PRNP,SOD2,AKT1,LDLR,ELANE,FAS,SNCA

Positive regulation of cellular catabolic process	11	413	6.55E-08	SIRT1,APOA1,APOE,APP,CASP3,SIRT6,IGF1,IL6,TNF,AKT1,SNCA
Regulation of amyloid precursor protein catabolic process	6	44	6.66E-08	APOE,APP,CASP3,IGF1,TNF,PRNP
Positive regulation of lipid biosynthetic process	7	86	6.76E-08	APOA1,APOE,IL1A,PTGS2,TNF,AKT1,LDLR
Positive regulation of protein kinase activity	11	416	6.96E-08	SIRT1,IL18,APP,IGF1,CDKN1A,ADIPOQ,TNF,PRNP,AKT1,ELANE,SNCA
Positive regulation of inflammatory response	8	145	7.10E-08	SERPINE1,IL18,APP,PTGS2,IL6,TNF,LDLR,SNCA
Blood vessel morphogenesis	11	419	7.43E-08	SIRT1,SERPINE1,CCL2,APOB,APOE,IL18,NOS3,CASP8,PTGS2,AKT1,LDLR
Negative regulation of inflammatory response	8	147	7.80E-08	APOA1,APOE,SOD1,ALOX5,IGF1,ADIPOQ,LDLR,ELANE
Cellular response to UV	7	90	8.87E-08	SIRT1,TP53,CASP9,PARP1,PTGS2,MMP9,CDKN1A
Negative regulation of cellular component organization	13	691	9.02E-08	APOA1,APOE,TP53,APP,PARP1,BCL2L1,IGF1,ADIPOQ,TNF,PRNP,AKT1,LDLR,SNCA
Cellular oxidant detoxification	7	91	9.46E-08	GSR,APOE,SOD1,ALB,NOS3,PTGS2,SOD2
Positive regulation of biosynthetic process	20	2080	1.07E-07	SIRT1,CCL2,APOA1,APOE,CYBA,IL1A,TP53,IL18,APP,NOS3,TERT,PARP1,PTGS2,IGF1,IL6,TNF,SOD2,AKT1,LDLR,SNCA
Cellular response to biotic stimulus	9	232	1.09E-07	SERPINE1,CCL2,IL1A,TP53,IL18,NOS3,IL6,TNF,AKT1

Response to amyloid-beta	6	49	1.14E-07	APP,PARP1,MMP9,IGF1,TNF,PRNP
Regulation of system process	12	567	1.19E-07	APOA1,APOE,CYBA,IL1A,SOD1,APP,NOS3,PARP1,PTGS2,IGF1,ADIPOQ,TNF
Positive regulation of immune system process	14	874	1.35E-07	SIRT1,SERPINE1,CCL2,IL1A,IL18,APP,KLK3,CASP8,IGF1,CDKN1A,IL6,TNF,AKT1,ELANE
Regulation of cellular metabolic process	31	5681	1.47E-07	SIRT1,APOA1,APOE,CYBA,IL1A,TP53,SOD1,IL18,APP,NOS3,TERT,CASP3,SIRT6,PARP1,PTGS2,FASLG,MMP9,ALOX5,BCL2L1,IGF1,CDKN1A,IL6,ADIPOQ,TNF,PRNP,SOD2,AKT1,LDLR,ELANE,FAS,SNCA
Regulation of monooxygenase activity	6	52	1.56E-07	APOE,IL1A,TERT,TNF,AKT1,SNCA
Regulation of transmembrane transport	12	587	1.71E-07	CCL2,CYBA,APP,TERT,SIRT6,MMP9,IGF1,ADIPOQ,TNF,PRNP,AKT1,SNCA
Regulation of nitrogen compound metabolic process	31	5734	1.86E-07	SIRT1,SERPINE1,APOA1,APOE,IL1A,TP53,SOD1,IL18,APP,NOS3,TERT,CASP3,CASP9,SIRT6,CASP8,PARP1,PTGS2,FASLG,MMP9,IGF1,CDKN1A,IL6,ADIPOQ,TNF,PRNP,SOD2,AKT1,LDLR,ELANE,FAS,SNCA
Response to corticosteroid	8	167	1.91E-07	CYBA,CASP3,CASP9,PARP1,PTGS2,IL6,ADIPOQ,TNF
Regulation of oxidoreductase activity	7	102	1.91E-07	APOE,CYBA,IL1A,TERT,TNF,AKT1,SNCA
Regulation of glucose import	6	55	2.08E-07	TERT,SIRT6,IGF1,ADIPOQ,TNF,AKT1
Response to alcohol	9	252	2.09E-07	CYBA,SOD1,CASP8,PARP1,BCL2L1,ADIPOQ,TNF,SOD2,AKT1
Negative regulation of hydrolase activity	10	354	2.22E-07	SERPINE1,APOA1,APP,NOS3,PTGS2,MMP9,TNF,PRNP,AKT1,SNCA
Response to temperature stimulus	8	172	2.34E-07	IL1A,SOD1,NOS3,CASP8,PTGS2,IGF1,SOD2,AKT1

Plasma lipoprotein particle clearance	5	24	2.44E-07	APOB,APOA1,APOE,ADIPOQ,LDLR
Cellular response to tumor necrosis factor	8	175	2.63E-07	SIRT1,CCL2,APOB,CYBA,TP53,TNF,AKT1,FAS
Response to cadmium ion	6	59	2.98E-07	SOD1,TERT,MMP9,PRNP,SOD2,AKT1
Intracellular signal transduction	17	1518	3.06E-07	SIRT1,CCL2,APOE,TP53,IL18,APP,NOS3,CASP3,CASP9,CASP7,BCL2L1,IGF1,CDKN1A,TNF,PRNP,SOD2,AKT1
Positive regulation of lipid localization	7	111	3.18E-07	SIRT1,APOB,APOA1,APOE,IL1A,FASLG,ADIPOQ
Signal transduction	28	4714	3.53E-07	SIRT1,CCL2,APOA1,APOE,IL1A,TP53,IL18,APP,NOS3,CASP3,CASP9,ITGAL,CASP8,PARP1,FASLG,CASP7,MMP9,BCL2L1,IGF1,CDKN1A,IL6,ADIPOQ,TNF,PRNP,SOD2,AKT1,FAS,SNCA
Positive regulation of oxidoreductase activity	6	61	3.53E-07	APOE,CYBA,TERT,TNF,AKT1,SNCA
Extrinsic apoptotic signaling pathway	7	113	3.53E-07	IL1A,CASP8,FASLG,CASP7,BCL2L1,TNF,FAS
Positive regulation of leukocyte cell-cell adhesion	9	274	4.00E-07	CCL2,IL1A,IL18,ALOX5,IGF1,IL6,TNF,AKT1,ELANE
Regulation of protein transport	11	503	4.14E-07	APOE,IL1A,APP,SIRT6,PTGS2,ALOX5,IGF1,IL6,ADIPOQ,TNF,PRNP
Negative regulation of cellular metabolic process	20	2265	4.18E-07	SIRT1,APOE,TP53,APP,TERT,CASP3,SIRT6,PARP1,FASLG,BCL2L1,IGF1,CDKN1A,IL6,ADIPOQ,TNF,PRNP,AKT1,LDLR,ELANE,SNCA
Regulation of carbohydrate metabolic process	8	189	4.44E-07	SIRT1,TP53,APP,SIRT6,IGF1,ADIPOQ,AKT1,SNCA

Cellular response to reactive oxygen species	7	118	4.54E-07	SIRT1,SOD1,NOS3,MMP9,IL6,SOD2,AKT1
Regulation of oxidative stress-induced intrinsic apoptotic signaling pathway	5	28	4.54E-07	SIRT1,SOD1,PARP1,SOD2,AKT1
Regulation of protein serine/threonine kinase activity	10	386	4.56E-07	SIRT1,APOE,APP,CASP3,CDKN1A,ADIPOQ,TNF,AKT1,ELANE,SNCA
Regulation of blood pressure	8	191	4.73E-07	CYBA,SOD1,NOS3,KLK3,PTGS2,ADIPOQ,TNF,SOD2
Regulation of biological process	41	11655	4.77E-07	SIRT1,GSR,SERPINE1,CCL2,APOB,APOA1,APOE,CYBA,IL1A,TP53,SOD1,IL18,APP,ALB,NOS3,TERT,CASP3,KLK3,CASP9,SIRT6,ITGAL,CASP8,PARP1,PTGS2,FASLG,CASP7,MMP9,ALOX5,BCL2L1,IGF1,CDKN1A,IL6,ADIPOQ,TNF,PRNP,SOD2,AKT1,LDLR,ELANE,FAS,SNCA
Macrophage differentiation	5	29	5.22E-07	SIRT1,APP,CASP8,PARP1,MMP9
Positive regulation of peptidyl-tyrosine phosphorylation	8	194	5.26E-07	TP53,IL18,APP,IGF1,IL6,ADIPOQ,TNF,PRNP
Cellular response to lipopolysaccharide	8	195	5.45E-07	SERPINE1,CCL2,IL1A,IL18,NOS3,IL6,TNF,AKT1
Cellular response to metal ion	8	198	6.05E-07	SOD1,APP,PARP1,PTGS2,MMP9,PRNP,AKT1,SNCA
Tube morphogenesis	12	669	6.22E-07	SIRT1,SERPINE1,CCL2,APOB,APOE,IL18,NOS3,CASP3,CASP8,PTGS2,AKT1,LDLR
Regulation of steroid biosynthetic process	6	70	6.98E-07	SIRT1,APOB,APOE,IL1A,SOD1,TNF

Developmental process	30	5657	7.23E-07	SIRT1,SERPINE1,CCL2,APOB,APOA1,APOE,IL1A,TP53,SOD1,IL18,APP,NOS3,CASP3,CASP9,SIRT6,CASP8,PARP1,PTGS2,FASLG,CASP7,MMP9,BCL2L1,IGF1,CDKN1A,IL6,ADIPOQ,TNF,SOD2,AKT1,LDLR
Negative regulation of cell differentiation	12	685	7.89E-07	SIRT1,IL1A,TP53,IL18,APP,MMP9,IGF1,IL6,ADIPOQ,TNF,SOD2,LDLR
Cellular response to peptide	9	302	8.28E-07	CYBA,TP53,APP,PARP1,IGF1,ADIPOQ,TNF,PRNP,AKT1
Regulation of cellular process	40	11025	8.29E-07	SIRT1,GSR,SERPINE1,CCL2,APOB,APOA1,APOE,CYBA,IL1A,TP53,SOD1,IL18,APP,ALB,NOS3,TERT,CASP3,CASP9,SIRT6,ITGAL,CASP8,PARP1,PTGS2,FASLG,CASP7,MMP9,ALOX5,BCL2L1,IGF1,CDKN1A,IL6,ADIPOQ,TNF,PRNP,SOD2,AKT1,LDLR,ELANE,FAS,SNCA
Regulation of generation of precursor metabolites and energy	7	131	8.41E-07	TP53,APP,SIRT6,IGF1,TNF,AKT1,SNCA
Response to xenobiotic stimulus	10	422	9.66E-07	CYBA,TP53,SOD1,CASP3,PTGS2,ADIPOQ,TNF,PRNP,SOD2,SNCA
Hematopoietic or lymphoid organ development	12	705	1.05E-06	SIRT1,TP53,SOD1,APP,CASP3,CASP9,CASP8,PARP1,MMP9,IL6,TNF,SOD2
Regulation of cholesterol metabolic process	5	35	1.14E-06	APOB,APOA1,APOE,SOD1,LDLR
Cellular response to cytokine stimulus	12	711	1.15E-06	SIRT1,CCL2,APOB,CYBA,IL1A,TP53,IL18,FASLG,IL6,TNF,AKT1,FAS
Regulation of lipid transport	7	139	1.21E-06	SIRT1,APOA1,APOE,IL1A,FASLG,ADIPOQ,AKT1
Tube development	13	880	1.22E-06	SIRT1,SERPINE1,CCL2,APOB,APOE,IL18,NOS3,CASP3,CASP8,PTGS2,TNF,AKT1,LDLR
Negative regulation of extrinsic apoptotic	5	36	1.28E-06	IL1A,TERT,BCL2L1,TNF,AKT1

signaling pathway in absence of ligand				
Regulation of amyloid-beta formation	5	37	1.43E-06	APOE,CASP3,IGF1,TNF,PRNP
Regulation of protein stability	9	328	1.57E-06	SIRT1,APOA1,TP53,TERT,CASP3,SIRT6,IGF1,PRNP,SNCA
Regulation of leukocyte migration	8	231	1.76E-06	SERPINE1,CCL2,IL1A,APP,IL6,TNF,AKT1,ELANE
Positive regulation of hydrolase activity	11	589	1.76E-06	SIRT1,CCL2,APOA1,APP,CASP9,CASP8,FASLG,TNF,AKT1,FAS,SNCA
Positive regulation of response to external stimulus	10	453	1.77E-06	SERPINE1,CYBA,IL18,APP,KLK3,PTGS2,IL6,TNF,LDLR,SNCA
Lipopolysaccharide-mediated signaling pathway	5	39	1.78E-06	CCL2,IL18,NOS3,TNF,AKT1
Regulation of anatomical structure morphogenesis	13	920	1.95E-06	SIRT1,SERPINE1,CCL2,APOA1,IL1A,NOS3,TERT,KLK3,SIRT6,FASLG,ALOX5,IL6,TNF
Behavior	11	598	1.98E-06	APOE,TP53,SOD1,APP,CASP3,PTGS2,PRNP,SOD2,AKT1,LDLR,SNCA
Negative regulation of proteolysis	9	339	1.98E-06	SERPINE1,TP53,APP,PTGS2,MMP9,TNF,PRNP,AKT1,SNCA
Response to copper ion	5	40	1.98E-06	IL1A,SOD1,APP,PRNP,SNCA
Regulation of nitric-oxide synthase activity	5	40	1.98E-06	APOE,IL1A,TERT,TNF,AKT1
Cellular response to amyloid-beta	5	40	1.98E-06	APP,PARP1,IGF1,TNF,PRNP

Positive regulation of protein localization	10	461	2.01E-06	IL1A,APP,TERT,SIRT6,PARP1,PTGS2,IGF1,TNF,PRNP,AKT1
Negative regulation of neuron apoptotic process	7	153	2.12E-06	CCL2,APOE,SOD1,TERT,BCL2L1,SOD2,SNCA
Response to hormone	12	762	2.24E-06	SIRT1,APOB,CYBA,NOS3,CASP3,CASP9,PARP1,PTGS2,IL6,ADIPOQ,TNF,AKT1
Negative regulation of endopeptidase activity	8	240	2.24E-06	SERPINE1,APP,PTGS2,MMP9,TNF,PRNP,AKT1,SNCA
Positive regulation of lipid transport	6	89	2.37E-06	SIRT1,APOA1,APOE,IL1A,FASLG,ADIPOQ
Cholesterol homeostasis	6	89	2.37E-06	SIRT1,APOB,APOA1,APOE,IL18,LDLR
Regulation of blood vessel endothelial cell migration	6	90	2.51E-06	SIRT1,APOE,NOS3,PTGS2,TNF,AKT1
Regulation of cellular carbohydrate metabolic process	7	158	2.57E-06	SIRT1,TP53,SIRT6,IGF1,ADIPOQ,AKT1,SNCA
Regulation of mononuclear cell proliferation	8	246	2.63E-06	IL1A,IL18,CASP3,BCL2L1,IGF1,CDKN1A,IL6,PRNP
Positive regulation of nitric oxide biosynthetic process	5	43	2.63E-06	APP,PTGS2,TNF,SOD2,AKT1
Regulation of purine nucleotide metabolic process	6	92	2.80E-06	APP,NOS3,SIRT6,PARP1,IGF1,SNCA
Positive regulation of cellular biosynthetic process	18	2041	2.86E-06	SIRT1,APOA1,APOE,IL1A,TP53,IL18,APP,NOS3,TERT,PARP1,PTGS2,IGF1,IL6,TNF,SOD2,AKT1,LDLR,SNCA

Positive regulation of cell adhesion	10	485	3.06E-06	CCL2,APOA1,IL1A,IL18,ALOX5,IGF1,IL6,TNF,AKT1,ELANE
Regulation of fatty acid metabolic process	6	94	3.12E-06	SIRT1,APOA1,PTGS2,ADIPOQ,AKT1,SNCA
Regulation of interleukin-1 beta production	6	95	3.29E-06	APOA1,APP,CASP8,IGF1,IL6,TNF
Regulation of chemokine production	6	96	3.47E-06	IL18,APP,IL6,ADIPOQ,TNF,ELANE
Regulation of mitotic cell cycle	10	493	3.49E-06	SIRT1,CCL2,IL1A,TP53,APP,TERT,IGF1,CDKN1A,TNF,AKT1
Regulation of epithelial cell proliferation	9	366	3.49E-06	SIRT1,CCL2,APOE,CYBA,SIRT6,ALOX5,IGF1,TNF,AKT1
Negative regulation of phosphorylation	9	368	3.64E-06	SIRT1,APOE,CASP3,SIRT6,CDKN1A,ADIPOQ,PRNP,AKT1,SNCA
Cytokine-mediated signaling pathway	9	369	3.70E-06	SIRT1,CCL2,IL1A,TP53,IL18,IL6,TNF,AKT1,FAS
Regulation of release of cytochrome c from mitochondria	5	47	3.78E-06	TP53,MMP9,BCL2L1,IGF1,AKT1
Response to heat	6	98	3.81E-06	IL1A,SOD1,NOS3,PTGS2,IGF1,AKT1
Replicative senescence	4	16	3.82E-06	SERPINE1,TP53,TERT,CDKN1A
Negative regulation of miRNA maturation	4	16	3.82E-06	TP53,TERT,IL6,TNF
Regulation of carbohydrate biosynthetic process	6	99	4.00E-06	SIRT1,SIRT6,IGF1,ADIPOQ,AKT1,SNCA

Cellular response to lipid	10	502	4.00E-06	SERPINE1,CCL2,IL1A,IL18,NOS3,CASP9,IL6,TNF,AKT1,LDLR
Myeloid cell differentiation	8	263	4.05E-06	SIRT1,APP,CASP3,CASP9,CASP8,PARP1,MMP9,TNF
Vasodilation	5	48	4.08E-06	APOE,SOD1,NOS3,TNF,SOD2
Regulation of binding	9	375	4.11E-06	APOE,APP,TERT,PARP1,MMP9,IGF1,CDKN1A,ADIPOQ,AKT1
Regulation of T cell activation	9	376	4.19E-06	CCL2,IL1A,SOD1,IL18,CASP3,IGF1,IL6,PRNP,AKT1
Hemopoiesis	11	655	4.36E-06	SIRT1,TP53,APP,CASP3,CASP9,CASP8,PARP1,MMP9,IL6,TNF,SOD2
Regulation of lymphocyte activation	10	508	4.36E-06	CCL2,IL1A,SOD1,IL18,CASP3,IGF1,CDKN1A,IL6,PRNP,AKT1
Negative regulation of intrinsic apoptotic signaling pathway	6	101	4.37E-06	SIRT1,PTGS2,MMP9,BCL2L1,SOD2,AKT1
Response to antibiotic	5	49	4.41E-06	TP53,SOD1,CASP3,CASP9,CASP8
Response to carbohydrate	7	175	4.56E-06	APOB,CYBA,IL1A,CASP3,PTGS2,ADIPOQ,SOD2
Execution phase of apoptosis	5	50	4.81E-06	CASP3,CASP9,CASP8,CASP7,AKT1
Regulation of cellular amide metabolic process	10	516	4.95E-06	APOE,TP53,APP,CASP3,IGF1,IL6,TNF,PRNP,AKT1,SNCA
Regulation of glucose metabolic process	6	104	5.06E-06	SIRT1,TP53,SIRT6,IGF1,ADIPOQ,AKT1
Regulation of phagocytosis	6	104	5.06E-06	CCL2,APOA1,CYBA,SOD1,ADIPOQ,TNF
Small molecule metabolic process	16	1645	5.12E-06	APOB,APOA1,APOE,TP53,APP,NOS3,SIRT6,PARP1,PTGS2,ALOX5,IGF1,ADIPOQ,TNF,AKT1,LDLR,SNCA
Regulation of MAPK cascade	11	668	5.14E-06	CCL2,APOE,IL1A,SOD1,APP,IGF1,IL6,ADIPOQ,TNF,ELANE,FAS

Negative regulation of intracellular signal transduction	10	519	5.14E-06	SIRT1,APOE,CASP8,PTGS2,MMP9,BCL2L1,ADIPOQ,PRNP,SOD2,AKT1
Regulation of endothelial cell apoptotic process	5	51	5.16E-06	SERPINE1,CCL2,TERT,FASLG,TNF
Negative regulation of transferase activity	8	274	5.21E-06	SIRT1,APOE,TP53,CASP3,CDKN1A,ADIPOQ,AKT1,SNCA
Regulation of epithelial cell apoptotic process	6	106	5.53E-06	SERPINE1,CCL2,TERT,FASLG,IL6,TNF
Negative regulation of fat cell differentiation	5	52	5.60E-06	SIRT1,IL6,ADIPOQ,TNF,SOD2
Negative regulation of smooth muscle cell proliferation	5	53	6.11E-06	APOE,NOS3,CDKN1A,ADIPOQ,SOD2
Negative regulation of mitochondrion organization	5	54	6.64E-06	TP53,APP,BCL2L1,IGF1,AKT1
Positive regulation of peptidyl-serine phosphorylation	6	110	6.72E-06	APP,PTGS2,IL6,TNF,AKT1,SNCA
Positive regulation of cell activation	9	401	6.73E-06	CCL2,IL1A,IL18,APP,IGF1,CDKN1A,IL6,TNF,AKT1
Blood circulation	9	403	6.98E-06	APOE,CYBA,SOD1,NOS3,KLK3,PTGS2,ADIPOQ,TNF,SOD2
Regulation of DNA metabolic process	10	541	7.26E-06	SIRT1,TP53,CASP3,SIRT6,PARP1,CDKN1A,IL6,ADIPOQ,TNF,AKT1
Positive regulation of cellular component organization	13	1049	7.26E-06	SERPINE1,APOA1,APOE,IL1A,TP53,APP,SIRT6,FASLG,MMP9,IGF1,IL6,TNF,SNCA
Necroptotic process	4	20	7.56E-06	TP53,FASLG,TNF,FAS

Negative regulation of protein serine/threonine kinase activity	6	113	7.66E-06	SIRT1,APOE,CASP3,CDKN1A,ADIPOQ,AKT1
Regulation of ion transport	11	700	7.76E-06	CCL2,CYBA,IL1A,APP,NOS3,PTGS2,MMP9,TNF,PRNP,AKT1,SNCA
Regulation of vesicle-mediated transport	10	551	8.42E-06	SERPINE1,CCL2,APOA1,APOE,CYBA,SOD1,APP,ADIPOQ,TNF,SNCA
Glucose homeostasis	7	196	8.85E-06	SIRT1,CYBA,SIRT6,ALOX5,IL6,ADIPOQ,AKT1
Regulation of cell cycle process	11	716	9.53E-06	SIRT1,CCL2,IL1A,TP53,APP,TERT,BCL2L1,IGF1,CDKN1A,TNF,AKT1
